# Reciprocal Connections between Parvalbumin-Expressing Cells and Adjacent Pyramidal Cells Are Regulated by Clustered Protocadherin γ

**DOI:** 10.1523/ENEURO.0250-23.2023

**Published:** 2023-10-26

**Authors:** Nanami Kawamura, Tomoki Osuka, Ryosuke Kaneko, Eri Kishi, Ryuon Higuchi, Yumiko Yoshimura, Takahiro Hirabayashi, Takeshi Yagi, Etsuko Tarusawa

**Affiliations:** 1 KOKORO-Biology Group, Laboratories for Integrated Biology, Graduate School of Frontier Biosciences, Osaka University, Suita, Osaka 565-0871, Japan; 2 Section of Visual Information Processing, National Institute for Physiological Sciences, National Institutes of Natural Sciences, Department of Physiological Sciences, The Graduate University for Advanced Studies, Okazaki, Aichi 444-8585, Japan; 3 Clinical Medicine Research Laboratory, Shonan University of Medical Sciences, Totsuka-ku, Yokohama 244-0806, Japan

**Keywords:** clustered protocadherin, electrophysiology, excitatory-inhibitory connections, parvalbumin, reciprocal connections, visual cortex

## Abstract

Functional neural circuits in the cerebral cortex are established through specific neural connections between excitatory and various inhibitory cell types. However, the molecular mechanisms underlying synaptic partner recognition remain unclear. In this study, we examined the impact of clustered protocadherin-γ (*cPcdhγ*) gene deletion in parvalbumin-positive (PV^+^) cells on intralaminar and translaminar neural circuits formed between PV^+^ and pyramidal (Pyr) cells in the primary visual cortex (V1) of male and female mice. First, we used whole-cell recordings and laser-scan photostimulation with caged glutamate to map excitatory inputs from layer 2/3 to layer 6. We found that *cPcdhγ*-deficient PV^+^ cells in layer 2/3 received normal translaminar inputs from Pyr cells through layers 2/3–6. Second, to further elucidate the effect on PV^+^-Pyr microcircuits within intralaminar layer 2/3, we conducted multiple whole-cell recordings. While the overall connection probability of PV^+^-Pyr cells remained largely unchanged, the connectivity of PV^+^-Pyr was significantly different between control and PV^+^-specific *cPcdhγ*-conditional knock-out (*PV-cKO*) mice. In control mice, the number of reciprocally connected PV^+^ cells was significantly higher than PV^+^ cells connected one way to Pyr cells, a difference that was not significant in *PV-cKO* mice. Interestingly, the proportion of highly reciprocally connected PV^+^ cells to Pyr cells with large unitary IPSC (uIPSC) amplitudes was reduced in *PV-cKO* mice. Conversely, the proportion of middle reciprocally connected PV^+^ cells to Pyr cells with large uIPSC amplitudes increased compared with control mice. This study demonstrated that *cPcdhγ* in PV^+^ cells modulates their reciprocity with Pyr cells in the cortex.

## Significance Statement

In the cerebral cortex, interneural connections, called reciprocal connections between excitatory and inhibitory cells are believed to facilitate rhythmic neural activity and proper information processing. The molecular mechanisms underlying these neural connections, however, remain unclear. This study demonstrates that the deletion of clustered protocadherin-γ (*cPcdhγ*) in parvalbumin (PV)-expressing cells disrupts the reciprocal connection between excitatory cells and PV^+^ cells within layer 2/3 of the visual cortex. *cPcdhγ* is a diverse membrane adhesion molecule with 22 isoforms, and has some different isoforms expressed in unique combinations in each cell. With its homophilic binding properties, *cPcdhγ* may serve as a cell recognition molecule to select binding partners between cells.

## Introduction

GABAergic interneurons in the cortex are classified into numerous types. Parvalbumin-positive (PV^+^) cells are a major inhibitory neuron type (Celio, 1986; Gonchar and Burkhalter, 1997; Kawaguchi and Kubota, 1997). Unlike other inhibitory neuronal cell types, PV^+^ cells exhibit a characteristic fast-spiking firing pattern (Kawaguchi, 1995) and synapse with proximal dendrites and cell bodies of their target excitatory neurons (Kawaguchi, 1995; Tamás et al., 1997). This configuration is believed to provide robust inhibition to target cells and regulate their firing timing (Cardin, 2018). Additionally, PV^+^ cells participate in regulating responses to visual stimuli (Atallah et al., 2012) and formation of γ waves (Sohal et al., 2009), which are associated with information processing (Singer, 1993). PV^+^ cells have input-output relationships with specific layers and neurons depending on the cell type. There is also selectivity in the binding relationships with neighboring excitatory and inhibitory cells, forming nonrandom microcircuits (Yoshimura and Callaway, 2005; Morishima et al., 2017), while PV^+^ cells target adjacent pyramidal (Pyr) cells nonspecifically (Packer and Yuste, 2011). However, the molecular mechanisms underlying the connection specificity between excitatory and inhibitory neurons remains unclear.

Previous studies have indicated that cell lineage plays a crucial role in establishing specific connections among excitatory cells. Excitatory cells originating from the same single radial glial cell preferentially form synaptic contacts in the sensory cortex (Packer and Yuste, 2011). Our previous findings demonstrated that clonal layer four excitatory cells in the barrel cortex preferentially form reciprocal connections compared with nonclonal layer four excitatory cells. Moreover, cell lineage-dependent reciprocal connections are significantly reduced on deletion of *cPcdh* (Tarusawa et al., 2016). Lv et al., also reported that patterned *cPcdh* expression in individual cells regulates the connectivity of clonal excitatory neurons in the cortex (Lv et al., 2022), further indicating that cPcdh is one of the potential molecules involved in intercellular target recognition. Cortical GABAergic interneurons originate from a different cell lineage compared with glutamatergic neurons (Marín and Müller, 2014). Deletion of *cPcdhγ* in cortical inhibitory cells is accompanied by cell death during early postnatal stages (Carriere et al., 2020; Leon et al., 2020), complicating our understanding of the specific function of *cPcdhγ* in neural circuit formation within cortical inhibitory neurons.

cPcdhs are cell adhesion membrane proteins that are highly expressed in CNS. In mice, the 58 *cPcdh* genes are organized into three gene clusters: *cPcdhα*, *cPcdhβ*, and *cPcdhγ* (Kohmura et al., 1998; Wu and Maniatis, 1999). It is now becoming clear that different neuronal cell types express different *cPcdh* isoforms (Chen et al., 2017; Katori et al., 2017; Mountoufaris et al., 2017; Gallerani and Au, 2020), and in neocortical excitatory neurons, approximately nine *cPcdh* isoforms are expressed (Lv et al., 2022). It is known that cPcdhs have homophilic binding properties, suggesting their involvement in differentiating between self and other neurons (Esumi et al., 2005; Kaneko et al., 2006; Schreiner and Weiner, 2010; Hirano et al., 2012; Lefebvre et al., 2012; Yagi, 2012, 2013; Mountoufaris et al., 2017; Rubinstein et al., 2017; Brasch et al., 2019; Goodman et al., 2022).

Deletion of *cPcdhαβγ* and *cPcdhγ* in mice results in deficits in left-right alternation of locomotor-like activity in spinal cords, while *cPcdhαβγ* knock-out (KO) hippocampal dissociated cultures exhibit abnormally correlated neural activity (Hasegawa et al., 2016, 2017), underscoring the role of *cPcdhs* in neural circuit formation.

In this study, we used PV-Cre mice to delete *cPcdhγ* in parvalbumin-positive (PV^+^) cells because PV expression starts around postnatal day 14 (P14) in visual cortex (Lecea et al., 1995), which is already past the peak of programmed interneuronal cell death (Wong et al., 2018). The PV^+^ cells lacking *cPcdhγ* normally survived at postnatal day 21 by avoiding early neonatal cell death. We show that the deletion of *cPcdhγ* in PV^+^ cells changes the unique connectivity of each PV^+^ cell with adjacent Pyr cells. These results suggest that *cPcdhs* regulate microcircuit formation between the excitatory and inhibitory cells.

## Materials and Methods

### Animals

Animal experiments in this study adhered to the Osaka University Experimental Regulations (approval number: FBS-14-002-1). Mice were kept in a 24-h cycle with 12 h of light and 12 h of darkness.

### Generation of mutant mice

Previously the *cPcdhγ CR1-floxed* mice were generated (Hoshino et al., 2023). A deletion allele lacking γ*CR1* exon (*ΔγCR1*-allele) was generated by Cre-mediated meiotic recombination by crossing with mice carrying *Sycp-Cre* transgene (Hasegawa et al., 2016).

PV^+^ inhibitory cell-specific *cPcdhγ* deletion mice were produced by crossing PV^+^ cell-specific Cre-expressing mice (*PV-ires-Cre*, B6;129P2-*Pvalbtm1(cre)Arbr/*J, The Jackson Laboratory, 008069) with *cPcdhγCR1 floxed* mice. To visualize PV^+^ inhibitory cells, *PV-Cre* and *cPcdh*γ*flox^PV-Cre^* mice were crossed with *Ai14* mice (*Ai14*, B6.Cg-*Gt(ROSA)26Sor^tm14(CAG-tdTomato)Hze^*/J, The Jackson Laboratory, 007914). We used PV-Cre hetero, and *Ai14* homo or hetero (*cPcdhγ^+/+; PV-Cre; Ai14/Ai14 or Ai14/+^*) mice for the control group and *cPcdhγCR1 flox* homo, *PV-Cre* hetero, *Ai14* homo, or hetero (*cPcdhγ^fl/fl; PV-Cre;Ai14/Ai14 or Ai14/+^*) mice for the conditional KO (*PV-cKO*) group, including both sexes.

### Protein extraction and Western blot analysis

A polyclonal antibody against the *cPcdhγ* constant region (pan-Pcdhγ) was developed in rabbits using *Pcdhγ*-A12 (NM_033595.4, amino acids 809–932). To express glutathione S-transferase (GST) fusion proteins, we subcloned the relevant cDNA fragments into the *pGEX4T-2* plasmid (GE Healthcare). Immunization and affinity purification were conducted as described earlier (Watanabe et al., 1998).

Brains from P0 mice of each genotype were homogenized in five volumes of H buffer [20 mm Tris-HCl (pH 8.0), 2 mm EDTA (pH 8.0), 0.32 m sucrose] supplemented with cOmplete protease inhibitor (Roche, catalog #04693116001). After centrifugation at 20,000 × *g* for 1 h at 4°C, the pellet (P2 fraction) was solubilized with S buffer [20 mm Tris-HCl (pH 8.0), 150 mm NaCl, 1.3% Triton X-100] with cOmplete protease inhibitor for 1 h at 4°C. After centrifugation at 20 000 × *g* for 1 h at 4°C, the supernatant was collected, and the protein concentration was determined by BCA protein assay (Thermo Fisher Scientific, catalog #23227). We used 40 μg of proteins for SDS-PAGE and blotted them onto a nitrocellulose membrane, followed by the standard procedure. The membranes were blocked with 2% nonfat dry milk in TBS-T (200 mm NaCl, 40 mm Tris, 0.1% Tween 20), then incubated with rabbit anti-pan-cPcdhγ and rabbit anti-β-actin (13E5, Cell Signaling Technology, catalog #4970S) overnight at 4°C. The next day, the membranes were washed with TBS-T and incubated with HRP-conjugated secondary antibody for 1 h at room temperature. Membranes were then washed and developed with GE HealthcareECL prime (Cytiva, catalog #RPN2232). All images were acquired and analyzed using the ChemiDoc MP Imaging System (Bio-Rad).

### *In situ* hybridization

Double fluorescent *in situ* hybridization (FISH) was performed as previously described (Hirano et al., 2012). To detect all the *Pcdhγ* (GenBank accession number NM_033584, nucleotides 2485–4383), *Parvalbumin* (*PV*; GenBank accession number NM_013645, nucleotides 16-866), and *vesicular glutamate transporter* (*Vglut1*, *Slc17a7*; GenBank accession number NM_182993, nucleotides 1655–2374) transcripts, *Pcdh-CR* cRNA was synthesized using a digoxigenin-UTP RNA labeling kit (Roche), while *PV* and *Vglut1* cRNA probes were synthesized using a fluorescein-UTP RNA labeling kit (Roche).

### Single-molecule fluorescence *in situ* hybridization (smFISH)

HCR amplification-based smFISH was performed following previous methods (Tsuneoka and Funato, 2020). P21 mice were anesthetized with isoflurane and subsequently decapitated, and their brains were quickly frozen in dry-ice-cooled hexane, before being stored at −80°C. The primary visual cortex (V1) was sectioned to a thickness of 10 μm and RNAs were detected by ISHpalette (Nepagene) using the manufacturer’s protocol. Twenty pairs of split-initiator DNA probes targeting the *Pcdhg* constant region and nine pairs of split-initiator DNA probes for *PV* were designed (Table 1). The probes were mixed for hybridization at a concentration of 20 nm in an HCR hybridization buffer and incubated at 37°C overnight. HCR amplification occurred using ISHpalette Short hairpin amplifier, SaraFluor 488-S45 for *Pcdhg*, and ISHpalette Short hairpin amplifier, ATTO647N-A161 for *PV* at 25°C for 2 h.

**Table 1:** List of primer sequences

For generating γCR1-floxed mice
gCR1A-F: 5′-GTCGACATAACTTCGTATAATGATTATGCTATAC GAAGTTATGTTAGTCTCCGGGTTGGTTC-3′
gCR1A-R: 5′-AAGCTTGTGCCTGGGTGAATCTTGCT-3′
gCR1B-F: 5′-GGTACCACAGGCCATTGTGAGGCATG-3′
gCR1B-R: 5′-GTCGACTTGCTGAAGAAACGGATCCC-3′
gCR1C-F: 5′-GGATCC CTCTTCCTTCTCCCAGCTAC-3′
gCR1C-R: 5′-GAGCTCACAGGTGTAAGGGATGGAGA-3′
gCR1D-F: 5′-AAGCTTTAGCCACTAAGCTTTCCTGGG-3′
gCR1D-R: 5′-GCTAGCAACAAAAGGGTAGCCCCCC-3′
gCR1E-F: 5′-GCTAGCGCCTTGAGAGCTGACTTCCA-3′
gCR1E-R: 5′-GCATGCCATGCCCCTTGAATCCAATTC-3′
ProbeA-F: 5′-TGCGCTTGGGCAAGTTAG-3′
ProbeA-R: 5′-CAGCCTATAAGAAGCGCTGC-3′
ProbeB-F: 5′-GACAGACAGAAACAGGGAGC-3′
ProbeB-R: 5′-CAGCAGTTGCCCAAGCTC-3′

### Cell counting of PV^+^ cells in the visual cortex

Mice were anaesthetized with isoflurane, perfused transcardially first with 25 mm PBS and then with 4% paraformaldehyde (PFA) in PB (pH 7.3). The brains, removed from the skull, were stored in PFA at 4°C overnight, then transferred to 25 mm PBS with 30% sucrose and kept at 4°C overnight. Cryostat sections were cut at 20-μm thickness, and slides were washed three times in 25 mm PBS for 5 min. For immunofluorescence, the slides were incubated with DAPI (1:20,000) for 20 min. They were then washed in 25 mm PBS for 5 min. Images were taken at 10× magnification using a KEYENCE BZ-9000 microscope. Cortical layers were identified based on their distinct cell densities. Each layer was enclosed in a 200-μm-wide square based on DAPI, and the number of cells within each layer was counted manually. Five fields of view were used per mouse for the analysis.

### Slice preparations

Mice were deeply anesthetized with isoflurane, and transcardially perfused with 5-ml ice-cold normal artificial CSF (ACSF; 126 mm NaCl, 3 mm KCl, 1.3 mm MgSO_4_, 1.2 mm NaH_2_PO_4_, 2.4 mm CaCl_2_, 26 mm NaHCO_3_, 10 mm glucose) containing 1 mm kynurenic acid, saturated with 95% O_2_ and 5% CO_2_. The brain was removed, and 300-μm thickness acute slices of the left hemisphere, containing the primary visual cortex (V1), were prepared in ice-cold ACSF without kynurenic acid using a micro slicer (VT1200S; Leica). To avoid cutting nerve fibers in V1, we cut the coronal slices parallel to the apical dendrites of Pyr cells. The slices were allowed to recover at 32–34°C for 1 h in an interface chamber through a normal ACSF oxygenated with a 95% O_2_ and 5% CO_2_. The slices were then submerged in normal ACSF oxygenated with a CO_2_/O_2_ gas mixture until recordings.

### Whole-cell recordings

All recordings were performed in the V1 monocular region. Fluorescent protein-expressing and-nonexpressing cells were identified under fluorescent and infrared differential interference contrast optics with an 40×, 0.8 NA water immersion lens (BX-50WI, Olympus). Normal ACSF oxygenated with a CO_2_/O_2_ gas mixture was flowed into the submerged chamber of the upright microscope. Glass patch pipettes (BF150-110-7.5, Sutter Instrument; 5–7 MΩ) were filled with a solution containing 130 mm K-gluconate, 8 mm KCl, 1 mm MgCl_2_, 0.6 mm EGTA, 10 mm HEPES, 3 mm MgATP, 0.5 mm Na_2_GTP, 10 mm Na-phosphocreatine, and 0.2% biocytin (pH 7.3 adjusted with KOH) for PV^+^ cells and for action potential recordings of Pyr cells; and 130 mm Cs-gluconate, 8 mm CsCl, 1 mm MgCl_2_, 0.6 mm EGTA, 3 mm MgATP, 0.5 mm Na_2_GTP, 10 mm HEPES, 10 mm Na-phosphocreatine, and 0.2% biocytin (pH 7.3 adjusted with CsOH) for uIPSC recordings in Pyr cells. Neurons with the soma located at least 50 μm below the cut surface of the slice were recorded. For the analysis, we selected cells with a series resistance of <25 MΩ. We did not use series resistance compensation. All recordings were conducted using a MultiClamp 700B (Molecular Devices) amplifier, and data were analyzed using pClamp11 software (Molecular Devices). PV^+^ cells were identified using tdTomato fluorescence. Pyr cells were identified by their triangle-like shape. After recording, the slices were fixed in 4% PFA in 0.1 m PB overnight at 4°C. After fixation, the recorded cells were visualized by staining with Alexa Fluor 488-conjugated streptavidin (code: 016-540-084, Jackson ImmunoResearch) to confirm the cell type and location. Upon rupture of the cell membrane, the resting membrane potential was immediately recorded. The firing pattern evoked by depolarizing current injections, was measured in the current-clamp mode. The input resistance was recorded by applying a 5-mV square wave pulse in the voltage-clamp mode. In the PV^+^ cells, the firing characteristics were analyzed using the first action potential generated by the depolarizing current injection. For the simultaneous whole-cell recording of PV^+^ cell–Pyr cell pairs, neurons located within 50 μm or 60–100 μm range were targeted. In all double or triple recordings, synaptic connections between neurons were assessed in bidirectionally by applying brief (2 ms) depolarizing voltage pulses (minimum 50 trials) to evoke action potentials in one cell, while recording synaptic responses in the other. The holding membrane potential of PV^+^ cells was set to –70 mV for uEPSCs recordings, and Pyr cells were set to 0 mV for uIPSCs recordings. The holding potentials were corrected for the liquid junction potential. To determine the paired pulse ratio, action potentials were induced twice at 50-ms intervals for uEPSCs and 100-ms intervals for uIPSCs.

### Laser photostimulation

The experiment was performed as previously described (Yoshimura and Callaway, 2005; Ishikawa et al., 2014). Photostimulation was achieved through focal photolysis of RuBi-caged glutamate (Tocris; 3574) using 10-ms flashes of blue light (440 nm) emitted by a diode laser (FV5-LDPSU, Olympus). The light was focused onto the slices using a 4×, 0.16 NA microscope objective. Laser power was adjusted to induce action potential in the recorded cell at one or two photostimulation spots in cell-attached mode. Photostimulation-evoked EPSCs were recorded in the layer 2/3 PV^+^ cells. Typically, photostimulations were applied to 9 × 22 spots surrounding the recorded cell at 4.5-s intervals in a quasi-random sequence. To demonstrate that the direct responses and uEPSCs could be distinguished, we recorded 9 × 20 uEPSCs of PV^+^ cells through photostimulation and introduced 1 μm tetrodotoxin (TTX) to block Na^+^ channels. The same conditions were repeated for recording 10 min later (Dantzker and Callaway, 2000).

### Analysis

#### Analysis for electrophysiology

The kinetics of action potentials, unitary EPSCs (uEPSCs), and unitary IPSCs (uIPSCs) were analyzed using Clampfit 11 (Molecular Devices). The action potential threshold was defined as the point where the membrane potential change rate surpassed 10 mV/ms. The amplitude measurements of uEPSCs and uIPSCs included failure events, but such events were excluded from the kinetic analysis of uEPSCs and uIPSCs. EPSCs induced by photostimulation were analyzed as described earlier (Yoshimura et al., 2005). The maps of photostimulation sites were aligned with laminar borders in fixed and stained tissues, and each site was assigned a laminar identity. The records from stimulus sites on the layer boundaries were analyzed as records of the layer where the stimulus site’s center point belonged. Electrical recordings obtained from the photostimulation were analyzed using MiniAnalysis (Synaptosoft) and other custom software written in MATLAB (RRID:SCR_001622). We measured peak time and amplitude of all EPSCs occurring 10-ms postphotostimulation for 120 ms. The count of EPSCs evoked by photostimulation included temporally overlapped EPSCs and isolated ones.

#### Dendritic morphologic analysis

The dendritic morphology of biocytin-filled PV^+^ cells in layer 2/3, which were visualized by streptavidin staining, was captured using a confocal microscope (SpinSR, Olympus) with a x40 lens. The thickness of the Z slices was set at 1-μm intervals, ranging from the slice surface to the point where the fibers were not visible (∼100 μm). Dendritic morphology was traced with the simple neurite tracer (SNT) plugin on ImageJ/FIJI (https://imagej.net/software/fiji/), and Sholl analysis was conducted using the SNT plugin. Concentric circles were positioned in 10-pixel (5.88 μm) steps from the cell body center.

#### Statistical analysis

Statistical analysis was performed using the Mann–Whitney *U* test, Welch’s *t* test or two-way ANOVA when two groups were compared. Dunn’s multiple comparisons test, Bonferroni’s multiple comparisons test and Holm–Šídák’s multiple comparisons test were also performed when more than two groups were compared. χ^2^ and Fisher exact tests were also performed for group comparison. A *p*-value of <0.05 was considered statistically significant.

## Results

### Normal development of *cPcdhγ^fl/fl; PV-Cre^* mice

**Figure 1.**
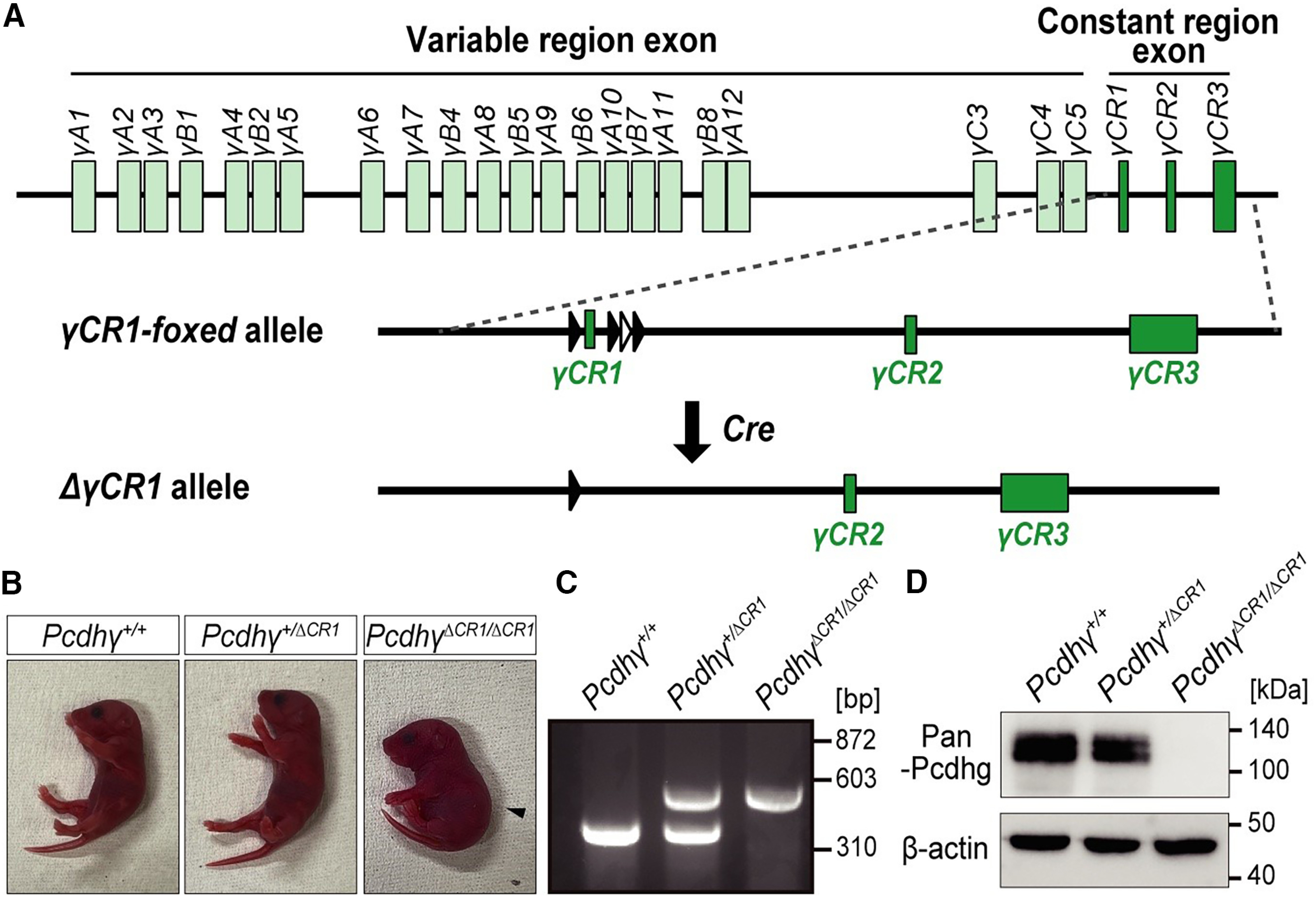
Confirmation of *cPcdhγ* deficiency by lacking *γCR1* exon. ***A***, Genomic structure of the *cPcdhγ* gene. The filled and open triangles represent *loxP and frt* sites, respectively. ***B***, Gross phenotypes of P0 neonatal pups. *cPcdhγ^ΔCR1/ΔCR1^* mutants had a hunched posture in most cases and died within 1 d after birth. *cPcdhγ^+/ΔCR1^* heterozygotes were survived. ***C***, PCR genotyping used to distinguish between wild-type (*cPcdhγ^+/+^*) and *cPcdhγ^ΔCR1/ΔCR1^* mutants. ***D***, Western blot analysis of the whole-brain lysate from P0 mouse brain. *cPcdhγ^ΔCR1/ΔCR1^* mutants did not express cPcdhγ protein. β-Actin was used as the loading control.

To investigate whether *cPcdhγ* in PV^+^ cells contributes to forming synaptic connections, *cPcdhγ* was specifically deleted in PV^+^ cells. Previous reports have shown that *cPcdhγ conditional KO* mice crossed with *gad2-Cre*, *Nkx2.1-Cre*, and *SST-Cre* mice display extensive inhibitory neural apoptosis in the cortex (Carriere et al., 2020; Leon et al., 2020), making it challenging to investigate the functional role of *cPcdhγ* in neural circuit formation. We also used *cPcdhγ flox* mice in which *γCR1* exon of *cPcdhγ* was floxed (Hoshino et al., 2023). To examine *cPcdhγ* deficiency by lacking *γCR1* exon, we produced the *γCR1* exon lacking allele by using Cre-induced mitotic recombination of *Sycp-Cre* transgenic mice (Fig. 1*A*,*C*). Homozygous pups were born but exhibited irregular breathing, repeated limb tremors, and died within 1 d of birth (Fig. 1*B*), similar phenotypes to previous reported *cPcdhγ* KO mice (Wang et al., 2002). And we also confirmed complete deletion of cPcdhγ proteins in the *γCR1* exon lacking homozygote brains (Fig. 1*D*). To produce conditional *cPcdhγ* lacking mice specifically in PV^+^ cells, we crossed between *cPcdhγ flox* and *PV-Cre* mice. PV expression starts around P14 in visual cortex, so that the Cre-induced *cPcdhγ* deletion of PV^+^ cells in *cPcdhγ^fl/fl; PV-Cre; Ai14/Ai14 or Ai14/+^* (*PV-cKO*) mice occur after this age. Therefore the PV^+^ cells in *PV-cKO* mice avoided cell death during early neonatal stages (<P14) in the cortex (Carriere et al., 2020; Leon et al., 2020).

**Figure 2.**
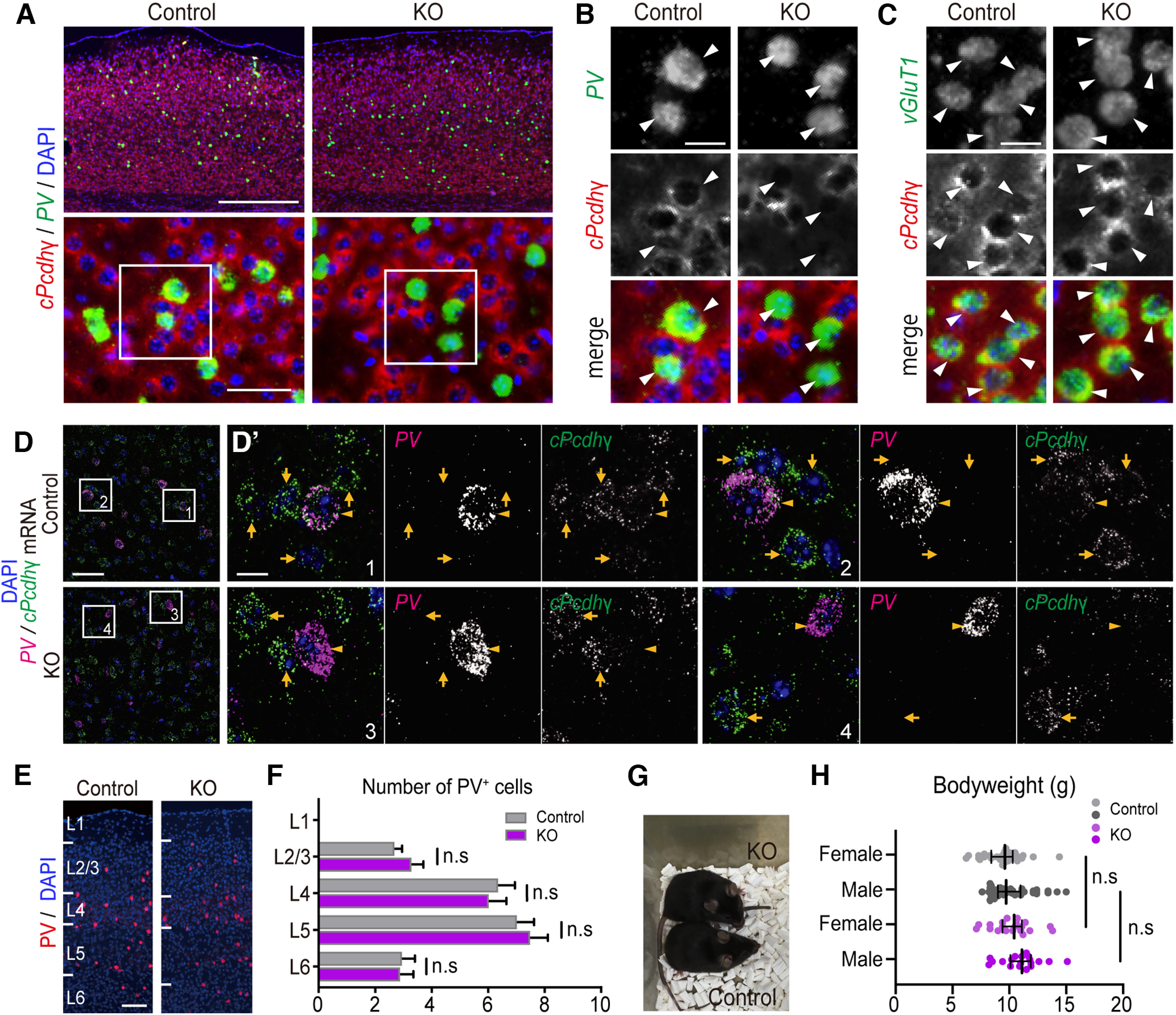
PV^+^ cell-specific *cPcdhγ* KO mice showed normal development. ***A***, Top, Low-magnification images of *cPcdhγ* (red) and *PV* (green) mRNA signals in the primary visual cortex at P21. Scale bar: 500 μm. Below, Higher-magnification image. Scale bar: 50 μm. ***B***, Magnified image of the white squares shown in ***A***. Arrowheads indicate cells exhibiting *PV* mRNA signals. Scale bar: 20 μm. ***C***, Same as ***B***, but representing image of *cPcdhγ* (red) and *vGluT1* (green) mRNA signals. Arrowheads indicate cells exhibiting a *vGluT1* mRNA signal. Scale bar: 20 μm. ***D***, Low-magnification images of smFISH of *PV* mRNA (magenta) and cPcdh*γ* mRNA (green) encoding constant exons common to all the *cPcdhγ* isoforms in the primary visual cortex at P21 of control (top) and *PV-cKO* (bottom). Scale bar: 50 μm. ***D’***, Magnified images of the white squares shown in ***D***. Arrowheads indicate cells exhibiting *PV* mRNA signals, and arrows indicate cells without *PV* mRNA signals. Scale bar: 10 μm. ***E***, Image of V1M obtained from P21 Control and *PV-cKO* mice. Scale bar: 100 μm. ***F***, Quantification of PV^+^ cells labeled with tdTomato in P21. Scatter bar plots summarize data, mean ± SEM from 12 sections. Two-way ANOVA with Sidak’s multiple comparisons test, *p* > 0.9999 (cont. L2/3 vs KO L2/3), *p* > 0.9999 (cont. L4 vs KO L4), *p* > 0.9999 (cont. L5 vs KO L5), *p* > 0.9999 (cont. L6 vs KO L6). Control: *n* = 3 mice; *PV-cKO*: *n* = 3 mice. ***G***, Picture of adult male mice at four months old. The upper panel is *PV-cKO* (*cPcdhγ^fl/fl; PV-Cre; Ai14/Ai14^*) and the lower panel is the control mouse (*cPcdhγ ^fl/fl; Ai14/Ai14^*). ***H***, Comparison of body weight in control and *PV-cKO* mice at P21–P26. Mann–Whitney *U* test. The bar indicates the median ± 95% CI: male cont. *n* = 37 mice; *PV-cKO n* = 29 mice, *p *=* *0.1457; female cont. *n* = 19 mice, *PV-cKO *=* *20 mice, *p *=* *0.1214. n.s. *p *>* *0.05.

To confirm the expression and deletion of *cPcdhγ* on P21, *in situ* hybridization for *cPcdhγ* mRNA detection was performed (Fig. 2*A–C*). The mRNA of *cPcdhγ* was detected in excitatory and PV^+^ cells in *cPcdhγ^+/+; PV-Cre; Ai14/Ai14 or Ai14/+^* mice (control mice), whereas the signals for *cPcdhγ* undetectable only in the PV^+^ cells of *cPcdhγ^fl/fl; PV-Cre; Ai14/Ai14 or Ai14/+^* mice (*PV-cKO* mice; Fig. 2*A–C*). Using a smFISH method with double *PV* and *cPcdhγ* probes, we also confirmed the PV^+^ cells specific *cPcdhγ* mRNA deletion in the primary visual cortex at P21 (Fig. 2*D*). Similar cell number and distribution of PV^+^ cells in the visual cortex were observed between control and *PV-cKO* mice at P21 (Fig. 2*E*,*F*), consistent with the previous study of Leon and colleagues (Leon et al., 2020; Carriere et al., 2020). *cPcdhγ−cKO* mice developed normally to adulthood, and their body weights were also similar to the control and *PV-cKO* mice (Fig. 2*G*,*H*).

### Deletion of *cPcdhγ* in PV^+^ cells does not affect the electrical membrane properties of PV^+^ and Pyr cells

**Figure 3.**
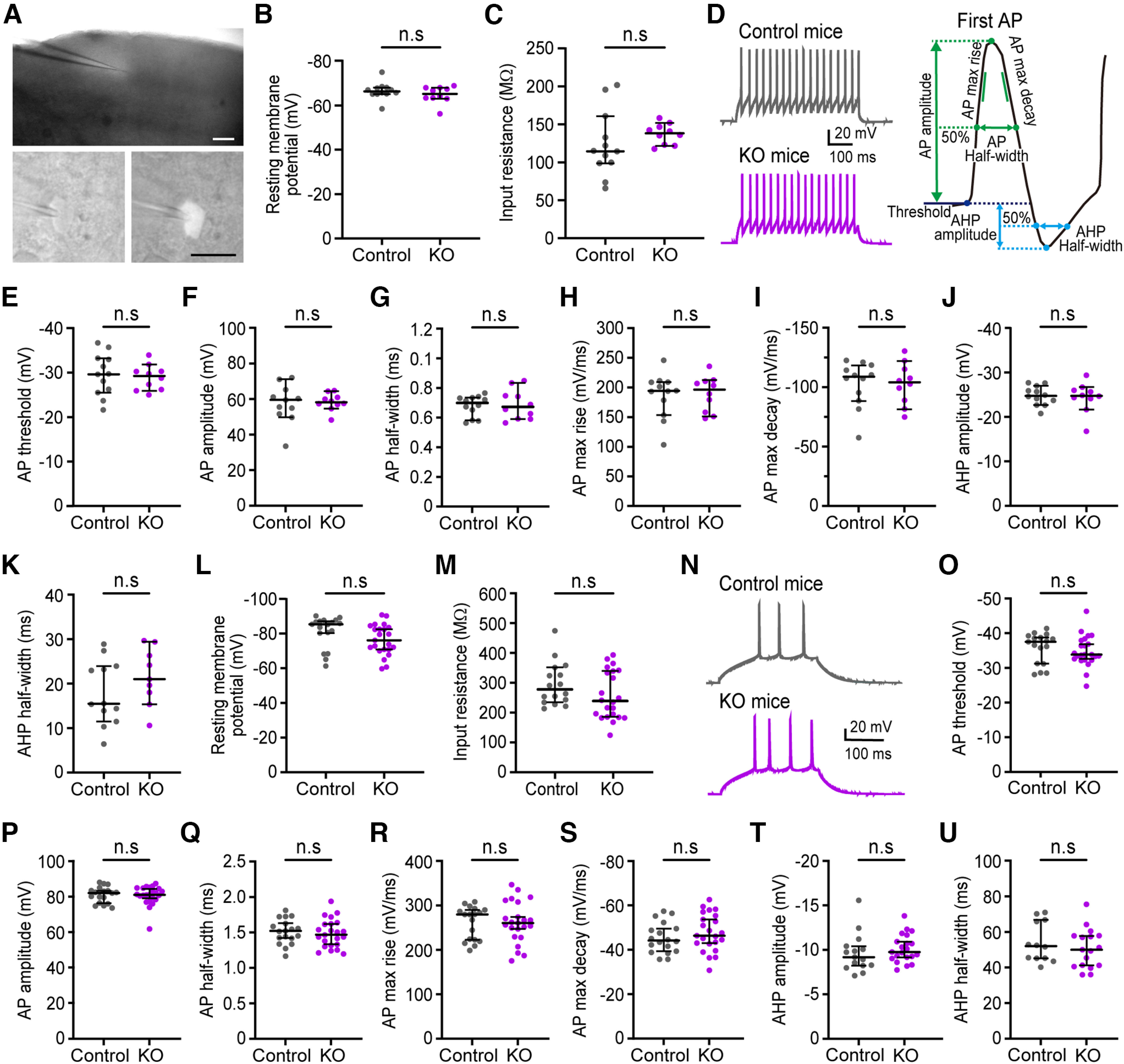
*PV-cKO* does not affect the electrical membrane properties of PV^+^ and Pyr cells. ***A*,** Image of a brain slice with a recording electrode in layer 2/3 of the V1 region (top). Scale bar: 200 μm. Lower images are recorded for td Tomato-positive cells. Scale bar: 20 μm. ***B–K***, Data were obtained from PV^+^ cells and (***L–U***) Pyr cells. ***B***, Resting membrane potential of PV^+^ cells (control: *n* = 12 cells; KO: *n* = 10 cells; *p *=* *0.4076). ***C***, Input resistance (control: *n* = 12 cells; KO: *n* = 10 cells; *p *=* *0.1072). ***D***, Left, Representative traces of firing patterns of action potentials (APs) evoked by depolarizing current injection (200 pA) of PV^+^ cells. Right, Schematic representation of AP and each measurement. ***E***, Threshold of AP (control, *n* = 12 cells; KO, *n* = 10 cells; *p *=* *0.8212). ***F***, Amplitude of AP (control: *n* = 11 cells; KO: *n* = 10 cells; *p *=* *0.9177). ***G***, Half-width of AP (control: *n* = 12 cells; KO: *n* = 10 cells; *p *=* *0.7223). ***H***, Maximum rise time of AP (control: *n* = 12 cells, KO: *n* = 10 cells; *p *=* *0.8718). ***I***, Maximum decay time of AP (control: *n* = 12 cells, KO: *n* = 10 cells; *p *=* *0.7713). ***J***, Amplitude of afterhyperpolarization (AHP; control: *n* = 12 cells, KO: *n* = 10 cells; *p *=* *0.9878). ***K***, Half-width of AHP (control: *n* = 12 cells; KO: *n* = 9 cells; *p *=* *0.2188). ***L***, Resting membrane potential of Pyr cells (control: *n* = 18 cells, KO: *n* = 23 cells; *p *=* *0.0536). ***M***, Input resistance (control: *n* = 16 cells; KO: *n* = 22 cells; *p *=* *0.1367). ***N***, Representative traces of AP firing patterns evoked by depolarizing current injection (200 pA) of Pyr cells. ***O***, Threshold of AP (control: *n* = 16 cells; KO: *n* = 22 cells; *p *=* *0.3563). ***P***, Amplitude of AP (control: *n* = 18 cells; KO: *n* = 23 cells; *p *>* *0.9999). ***Q***, Half-width of AP (control: *n* = 18 cells; KO: *n* = 23 cells; *p *=* *0.4905). ***R***, Maximum rise time of AP (control: *n* = 18 cells, KO: *n* = 23 cells; *p *=* *0.8455). ***S***, Maximum decay time of AP (control: *n* = 18 cells, KO: *n* = 23 cells; *p *=* *0.2674). ***T***, Amplitude of afterhyperpolarization (AHP; control: *n* = 15 cells; KO: *n* = 21 cells; *p *=* *0.2385). ***U***, AHP half-width (control: *n* = 12 cells; KO: *n* = 17 cells; *p *=* *0.3938). ***B***, ***C*, *E–L***, ***O*–*U***, Mann–Whitney *U* test, The bar indicates the median ± 95% CI value. ***B–M***, Control: *n* = 10 mice; *PV-cKO*: *n* = 9 mice. ***N–U***, Control: *n* = 3 mice; *PV-cKO*: *n* = 4 mice. n.s. *p *>* *0.05.

To evaluate the effect of *cPcdhγ* deletion of on the membrane properties of PV^+^ and Pyr cells in *PV-cKO* mice, whole-cell recordings were taken from PV^+^ cells, which were visualized using tdTomato (Fig. 3*A–K*), and Pyr cells (Fig. 3*L–U*) in layer 2/3 (L2/3) of the primary visual cortex at P21–P26. There were no significant differences in the resting membrane potential, input resistance, and action potential kinetics of PV^+^ cells and Pyr cells between control and *PV-cKO* mice. This suggests that *cPcdhγ* deletion in PV^+^ cells does not impact the electrical cell membrane properties.

### *cPcdhγ* in PV^+^ cells affect the dendritic morphology of PV^+^ cells

**Figure 4.**
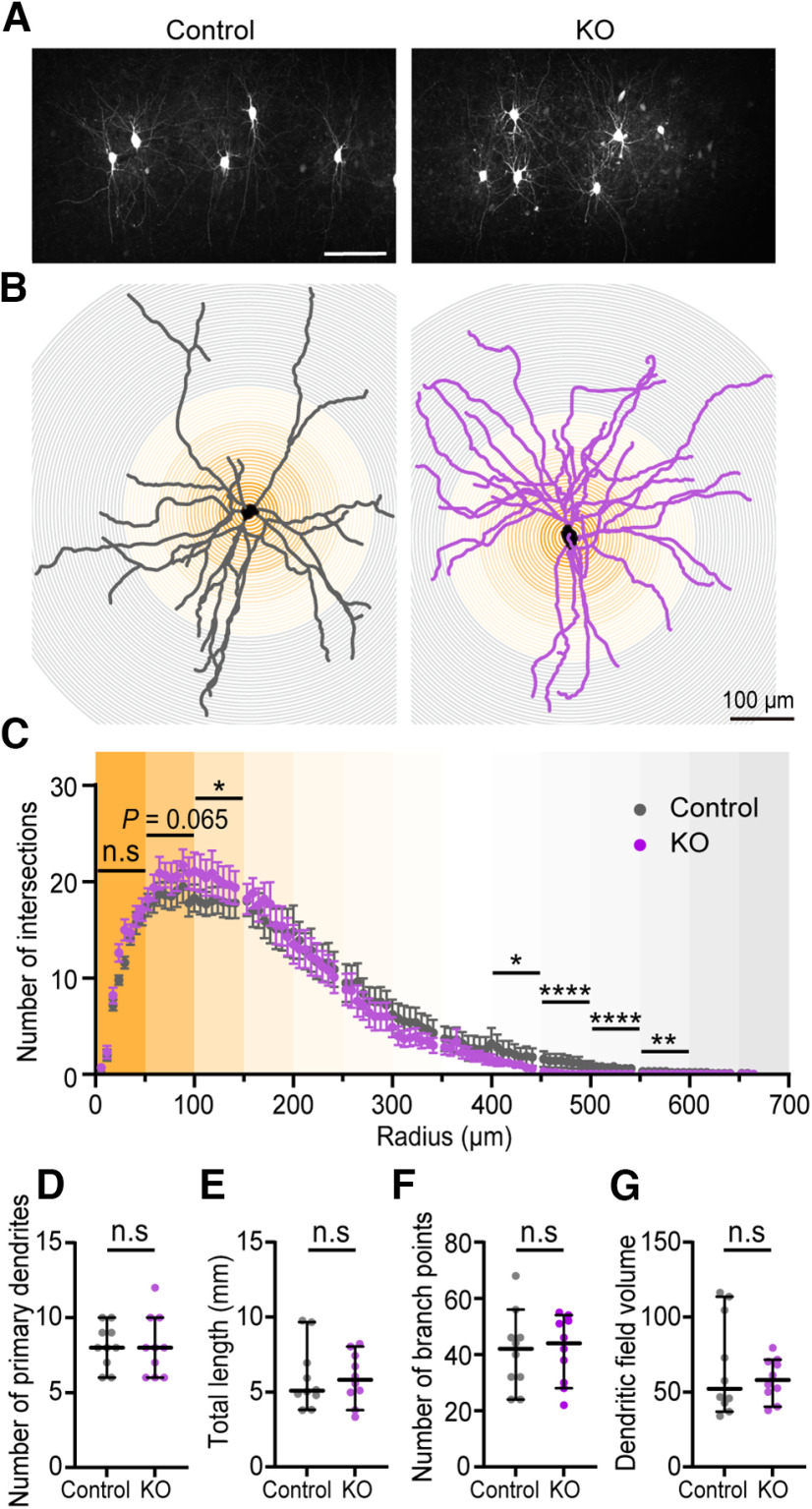
*cPcdhγ* deficient PV^+^ cells in L2/3 have increased dendritic complexity near the cell body. ***A***, Representative maximum Z-stack image of PV^+^ cells stained with biocytin in primary visual cortex. Scale bar: 100 μm. ***B***, Representative Sholl dendritic analysis of reconstructed biocytin-filled PV^+^ cells using a whole-cell patch clamp (left; control, right; *PV-cKO*). Centered on the cell body (shown in black), the regions (0–49, 50–99, 100–149, and 150–200 μm) are represented by color intensity (shown in orange). ***C***, Number of intersections are presented here. The bar indicates SEM. Two-way ANOVA with Holm–Šídák’s multiple comparisons test, *p *=* *0.8067. The numbers of crossing PV^+^ dendrites were significantly reduced in 100–149 μm (*p *=* *0.0454), 400–449 μm (*p *= 0.0469), 450–499 μm (*p *<* *0.0001), 500–549 μm (*p *<* *0.0001), 550–599 μm (*p *=* *0.0041) of *PV-cKO* compared with control. There were no significantly different in 0–49 μm (*p *=* *0.5129), 50–99 μm (*p *= 0.0651), 150–199 μm (*p *=* *0.5593), 200–249 μm (*p *=* *0.5593), 250–299 μm (*p *=* *0.4487), 300–349 μm (*p *=* *0.0789), 350–399 μm (*p *= 0.4716) in *PV-cKO* as compared with control. ***D***, Number of primary dendrites. ***E***, Total dendrite length. ***F***, Number of branch points. ***G***, Dendritic field area. ***D–G***, Bars indicate the median ± 95% CI value. Mann–Whitney *U* test, *p *=* *0.8214 (***D***), *p *=* *0.7959 (***E***), *p *=* *0.8975 (***F***), *p *>* *0.9999 (***G***). ***C–G***, Control: *n* = 10 cells; *PV-cKO*: *n* = 10 cells. Significance is indicated in the figures as follows: *p <* *0.05, **p <* *0.01, ****p <* *0.0001, n.s. *p *>* *0.05.

To identify the effect by *PV-cKO* on PV^+^ cell morphology, dendrite morphologic analysis of biocytin-filled PV^+^ cells was conducted (Fig. 4). We confirmed the cell type of the PV^+^ cells by visualizing the morphology with biocytin injection and found no chandelier cells (Fig. 4*A*). Compared with control PV^+^ cells, the complexity of dendrites within 50–150 μm from the soma was most significantly increased in *PV-cKO* PV^+^ cells, and longer dendrites beyond 450 μm from the soma were significantly reduced in *PV-cKO* PV^+^ cells (Fig. 4*B*,*C*). However, the number of primary dendrites, total dendritic length, number of branch points, and dendritic field area were similar between control and *PV-cKO* cells (Fig. 4*D–G*).

### Deletion of *cPcdhγ* in PV^+^ cells on the excitatory does not significant affect translaminar and intralaminar inputs

**Figure 5.**
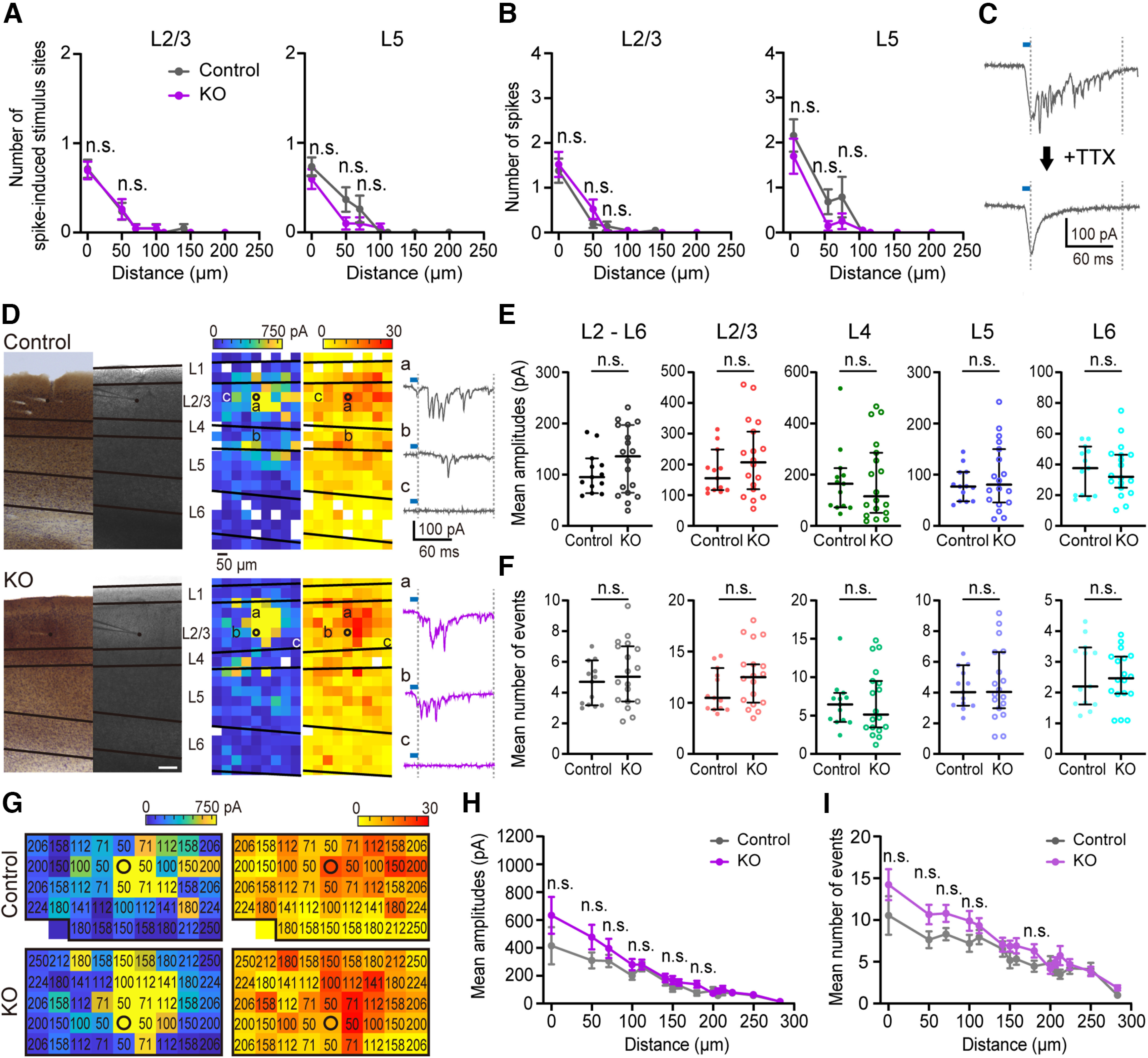
PV^+^ cells in *PV-cKO* mice receive same inputs from surrounding Pyr cells compared with control mice. ***A***, The number of stimulus sites where cell firing was induced in recorded Pyr cells at L2/3 (left) and L5 (right) by laser photostimulation and their distance from the recording site is presented here. The bar indicates SEM. ***B***, Number of spikes per laser photostimulation and their distance from the recording Pyr cells of L2/3 (left) and L5 (right), respectively. The bar indicates. ***C***, Representative example of same EPSC traces by photostimulation. Bottom, Ten minutes after the addition of TTX (bottom). The blue bar represents the laser exposure period. ***D***, Left, Image of a brain slice recorded from PV^+^ cells in a layer 2/3 V1 region (right) and cytochrome *c* oxidase and Nissl-stained image of the same slice as in the right (left). Scale bar: 100 μm. Right, Photostimulation-evoked EPSCs (EPSCs) recorded in layer 2/3 PV^+^ cells. Reconstructions of the locations of photostimulation sites (colored squares) relative to the locations of laminar borders and cell bodies of the recorded PV^+^ cells (open black circles) are shown. The white squares indicate no input. Left, The color of each square indicates the sum of the amplitudes of EPSCs that were observed in response to photostimulation at that site. Right, The color of each square indicates the number of EPSCs events observed in response to photostimulation at that site. The EPSC traces of the photostimulation at each spot (indicated by a, b, c) are presented on the right. The blue bar represents the laser exposure period. ***E***, The mean amplitude of photostimulation-evoked EPSCs for each layer is plotted. The bars indicate median ± 95% CI values. ***F***, Mean number of events induced by photostimulation-evoked excitatory responses. The bars indicate median ± 95% CI values. ***G***, L2/3 only input amplitude map (left) and input event map (right). The location of the recorded PV^+^ cell is indicated by an open black circle and the number in the squares indicates the distance from the recording cell. ***H***, Mean amplitude at each distance from the recorded PV^+^ cells. The bar indicates SEM. ***I***, Mean number of events at each distance from recorded PV^+^ cells. The bar indicates SEM (***A***, ***B***, ***H***, ***I***) Bonferroni’s multiple comparisons test. Significance is indicated in the figures as follows: *p <* *0.05, n.s. *p *>* *0.05. Refer to Table 2 for statistical information.

Because the complexity and the length of dendrites of PV^+^ cells were abnormal in *PV-cKO* mice (Fig. 4*B*,*C*), next we analyzed the neuronal connectivity between PV^+^ cells and Pyr cells in the whole visual cortex. To determine whether *cPcdhγ* deletion in L2/3 PV^+^ cells causes any changes in local excitatory inputs onto PV^+^ cells, we analyzed the laminar source and the strength of excitatory inputs to PV^+^ cells using laser scanning photostimulation with caged glutamate (Yoshimura et al., 2005; Ishikawa et al., 2014). RuBi-glutamate was uncaged using blue light for the photostimulation of cortical cells from L1 to L6. To confirm that the action potential induction of cortical neurons by photostimulation was comparable between the control and *PV-cKO* groups, loose patch-clamp recordings were made from L2/3 and L5 Pyr cells (Fig. 5*A*,*B*). The photostimulation-evoked action potentials were observed only when the locations of the recorded cell were stimulated, and no action potentials were induced at any other locations from L1 to L6 in both groups. The number of stimulation sites where action potentials were evoked by photostimulation in the recorded L2/3 and L5 Pyr cells (Fig. 5*A*), and the number of action potentials evoked by photostimulation at single stimulation sites in the recorded L2/3 and L5 Pyr cells (Fig. 5*B*), were not significantly different between the control and *PV-cKO* groups (Table 2). When photostimulation was applied near the PV^+^ cell body, evoked EPSCs and direct responses were observed. Figure 5*C* shows that the direct response of PV^+^ cells can be separated from photostimulation-evoked EPSCs inputs in temporal resolution (Dantzker and Callaway, 2000). Figure 5*D* presents representative spatial distributions of neurons presynaptic to the recorded PV^+^ cells, with color-coding for the number and amplitude of photostimulation-evoked EPSCs. L2/3 PV^+^ cells received excitatory inputs primarily from L2/3, L4, and moderately from L5 to L6 in both the control and *PV-cKO* groups (Fig. 5*E*,*F*), in accordance with previous reports (Dantzker and Callaway, 2000; Xu and Callaway, 2009). The *cPcdhγ*-deleted PV^+^ cells are no significant different from control mice in any layer (Fig. 5*E*,*F*). To verify more detailed excitatory input from L2/3 excitatory cells that are within the same layer, the input number and mean amplitudes were compared by distance from recording cell (Fig. 5*H*,*I*). As a result, no significant differences were found in *PV-cKO* groups (Table 2).

**Table 2:** Statistical information for the data shown in **Figure 5**

Figure 5*A*		
Group	Values	Number of animals
L2/3_Control		7 (*n* = 21 cells)
0 μm	0.714 ± 0.101	
50 μm	0.238 ± 0.095	
71 μm	0.048 ± 0.048	
100 μm	0.048 ± 0.048	
141 μm	0.048 ± 0.048	
150 μm	0 ± 0	
L2/3_KO		7 (*n* = 23 cells)
0 μm	0.698 ± 0.098	
50 μm	0.261 ± 0.113	
71 μm	0.043 ± 0.043	
100 μm	0 ± 0	
141 μm	0 ± 0	
150 μm	0 ± 0	
L5_Control		7 (*n* = 19 cells)
0 μm	0.737 ± 0.104	
50 μm	0.368 ± 0.137	
71 μm	0.263 ± 0.150	
100 μm	0 ± 0	
141 μm	0 ± 0	
150 μm	0 ± 0	
L5_KO		7 (*n* = 20 cells)
0 μm	0.600 ± 0.112	
50 μm	0.100 ± 0.069	
71 μm	0.100 ± 0.069	
100 μm	0.050 ± 0.050	
141 μm	0 ± 0	
150 μm	0 ± 0	

Comparison	*p*-value
L2/3_Control vs KO (two-way ANOVA)	0.8470
L2/3_Control (0 μm) vs KO (0 μm)	>0.9999
L2/3_Control (50 μm) vs KO (50 μm)	>0.9999
L2/3_Control (71 μm) vs KO (71 μm)	>0.9999
L2/3_Control (100 μm) vs KO (100 μm)	>0.9999
L2/3_Control (141 μm) vs KO (141 μm)	>0.9999
L5_Control vs KO (two-way ANOVA)	0.1417
L5_Control (0 μm) vs KO (0 μm)	>0.9999
L5_Control (50 μm) vs KO (50 μm)	0.6408
L5_Control (71 μm) vs KO (71 μm)	>0.9999
L5_Control (100 μm) vs KO (100 μm)	>0.9999

Figure 5*B*		
Group	Values	Number of animals
L2/3_Control		7 (*n* = 21 cells)
0 μm	1.318 ± 0.271	
50 μm	0.190 ± 0.088	
71 μm	0.143 ± 0.104	
100 μm	0.048 ± 0.048	
141 μm	0.048 ± 0.048	
150 μm	0 ± 0	
L2/3_KO		7 (*n* = 23 cells)
0 μm	1.522 ± 0.280	
50 μm	0.522 ± 0.217	
71 μm	0 ± 0	
100 μm	0.043 ± 0.043	
141 μm	0 ± 0	
150 μm	0 ± 0	
L5_Control		7 (*n* = 19 cells)
0 μm	2.158 ± 0.361	
50 μm	0.684 ± 0.276	
71 μm	0.789 ± 0.450	
100 μm	0 ± 0	
141 μm	0 ± 0	
150 μm	0 ± 0	
L5_KO		7 (*n* = 20 cells)
0 μm	1.700 ± 0.391	
50 μm	0.150 ± 0.109	
71 μm	0.250 ± 0.176	
100 μm	0.050 ± 0.050	
141 μm	0 ± 0	
150 μm	0 ± 0	

Comparison	*p*-value
L2/3_Control vs KO (two-way ANOVA)	0.6349
L2/3_Control (0 μm) vs KO (0 μm)	>0.9999
L2/3_Control (50 μm) vs KO (50 μm)	>0.9999
L2/3_Control (71 μm) vs KO (71 μm)	>0.9999
L2/3_Control (100 μm) vs KO (100 μm)	>0.9999
L2/3_Control (141 μm) vs KO (141 μm)	>0.9999
L5_Control vs KO (two-way ANOVA)	0.1493
L5_Control (0 μm) vs KO (0 μm)	0.8269
L5_Control (50 μm) vs KO (50 μm)	0.4796
L5_Control (71 μm) vs KO (71 μm)	0.4610
L5_Control (100 μm) vs KO (100 μm)	>0.9999
L5_Control (141 μm) vs KO (141 μm)	>0.9999
L5_Control (150 μm) vs KO (150 μm)	>0.9999

Figure 5*E*		
Group	Values	Number of animals
L2–6_Control	94.99	8 (*n* = 13 cells)
L2–6_KO	135.7	7 (*n* = 18 cells)
L2/3_Control	154.8	8 (*n* = 13 cells)
L2/3_KO	206.5	7 (*n* = 18 cells)
L4_Control	164.1	8 (*n* = 13 cells)
L4_KO	115.1	7 (*n* = 18 cells)
L5_Control	76.83	8 (*n* = 13 cells)
L5_KO	80.42	7 (*n* = 18 cells)
L6_Control	37.54	8 (*n* = 13 cells)
L6_KO	31.94	7 (*n* = 18 cells)

Comparison	Test used	*p*-value
L2–6_Control vs L2–6_KO	Mann–Whitney *U* test	0.4175
L2/3_Control vs L2/3_KO	Mann–Whitney *U* test	0.3949
L4_Control vs L4_KO	Mann–Whitney *U* test	0.8592
L5_Control vs L5_KO	Mann–Whitney *U* test	0.7674
L6_Control vs L6_KO	Mann–Whitney *U* test	0.8902

Figure 5*F*		
Group	Values	Number of animals
L2–6_Control	4.699	8 (*n* = 13 cells)
L2–6_KO	5.034	7 (*n* = 18 cells)
L2/3_Control	5.476	8 (*n* = 13 cells)
L2/3_KO	7.501	7 (*n* = 18 cells)
L4_Control	6.444	8 (*n* = 13 cells)
L4_KO	5.139	7 (*n* = 18 cells)
L5_Control	4.021	8 (*n* = 13 cells)
L5_KO	4.038	7 (*n* = 18 cells)
L6_Control	2.196	8 (*n* = 13 cells)
L6_KO	2.463	7 (*n* = 18 cells)

Comparison	Test used	*p*-value
L2–6_Control vs L2–6_KO	Mann–Whitney *U* test	0.3521
L2/3_Control vs L2/3_KO	Mann–Whitney *U* test	0.2416
L4_Control vs L4_KO	Mann–Whitney *U* test	0.8902
L5_Control vs L5_KO	Mann–Whitney *U* test	0.8283
L6_Control vs L6_KO	Mann–Whitney *U* test	0.7374

Figure 5*H*		
Group	Values	Number of animals
Control		7 (*n* = 13 cells)
0 μm	414.1 ± 133.8	
50 μm	309.7 ± 57.60	
71 μm	305.4 ± 44.35	
100 μm	201.0 ± 30.60	
112 μm	263.2 ± 33.16	
141 μm	160.4 ± 30.86	
150 μm	105.8 ± 21.63	
158 μm	129.3 ± 22.25	
180 μm	75.10 ± 18.93	
200 μm	91.46 ± 27.02	
206 μm	58.13 ± 11.20	
212 μm	77.15 ± 18.42	
224 μm	76.19 ± 16.01	
250 μm	58.35 ± 13.92	
283 μm	14.83 ± 0	
ΚΟ		7 (*n* = 18 cells)
0 μm	632.7 ± 132.3	
50 μm	476.5 ± 87.88	
71 μm	395.1 ± 69.73	
100 μm	279.2 ± 39.87	
112 μm	279.4 ± 35.57	
141 μm	183.0 ± 30.80	
150 μm	168.0 ± 29.71	
158 μm	151.8 ± 22.95	
180 μm	138.3 ± 24.81	
200 μm	73.83 ± 14.01	
206 μm	90.29 ± 17.91	
212 μm	104.6 ± 22.90	
224 μm	79.62 ± 13.33	
250 μm	63.68 ± 12.28	
283 μm	14.61 ± 1.088	

Comparison	*p*-value
Control vs KO (two-way ANOVA)	0.1810
Control (0 μm) vs KO (0 μm)	0.0762
Control (50 μm) vs KO (50 μm)	0.4269
Control (71 μm) vs KO (71 μm)	>0.9999
Control (100 μm) vs KO (100 μm)	>0.9999
Control (112 μm) vs KO (112 μm)	>0.9999
Control (141 μm) vs KO (141 μm)	>0.9999
Control (150 μm) vs KO (150 μm)	>0.9999
Control (158 μm) vs KO (158 μm)	>0.9999
Control (180 μm) vs KO (180 μm)	>0.9999
Control (200 μm) vs KO (200 μm)	>0.9999
Control (206 μm) vs KO (206 μm)	>0.9999

Figure 5*I*		
Group	Values	Number of animals
Control		7 (*n* = 13 cells)
0 μm	10.54 ± 2.306	
50 μm	7.641 ± 0.9982	
71 μm	8.308 ± 0.7301	
100 μm	7.212 ± 1.016	
112 μm	7.954 ± 0.8549	
141 μm	6.526 ± 0.9189	
150 μm	5.179 ± 0.9562	
158 μm	5.323 ± 0.6842	
180 μm	4.469 ± 0.6703	
200 μm	4.859 ± 0.8234	
206 μm	3.955 ± 0.5724	
212 μm	3.500 ± 0.5627	
224 μm	4.212 ± 0.6590	
250 μm	4.125 ± 0.7447	
283 μm	1.000 ± 0	
ΚΟ		7 (*n* = 18 cells)
0 μm	14.22 ± 1.846	
50 μm	10.65 ± 1.180	
71 μm	10.79 ± 1.012	
100 μm	9.898 ± 1.192	
112 μm	9.330 ± 0.9095	
141 μm	7.046 ± 0.8313	
150 μm	6.875 ± 0.6562	
158 μm	6.905 ± 0.9187	
180 μm	6.313 ± 0.8082	
200 μm	4.194 ± 0.6433	
206 μm	4.930 ± 0.6196	
212 μm	5.739 ± 1.147	
224 μm	4.694 ± 0.6442	
250 μm	3.833 ± 0.5829	
283 μm	1.875 ± 0.3146	

Comparison	*p*-value
Control vs KO (two-way ANOVA)	0.1118
Control vs KO (0 μm)	>0.9999
Control vs KO (50 μm)	0.6719
Control vs KO (71 μm)	0.6185
Control vs KO (100 μm)	>0.9999
Control vs KO (112 μm)	>0.9999
Control vs KO (141 μm)	>0.9999
Control vs KO (150 μm)	>0.9999
Control vs KO (158 μm)	>0.9999
Control vs KO (180 μm)	0.9869
Control vs KO (200 μm)	>0.9999
Control vs KO (206 μm)	>0.9999

### The deletion of *cPcdhγ* in PV^+^ cells increases the connection probability from PV^+^ to 50–100 μm apart Pyr cells

**Figure 6.**
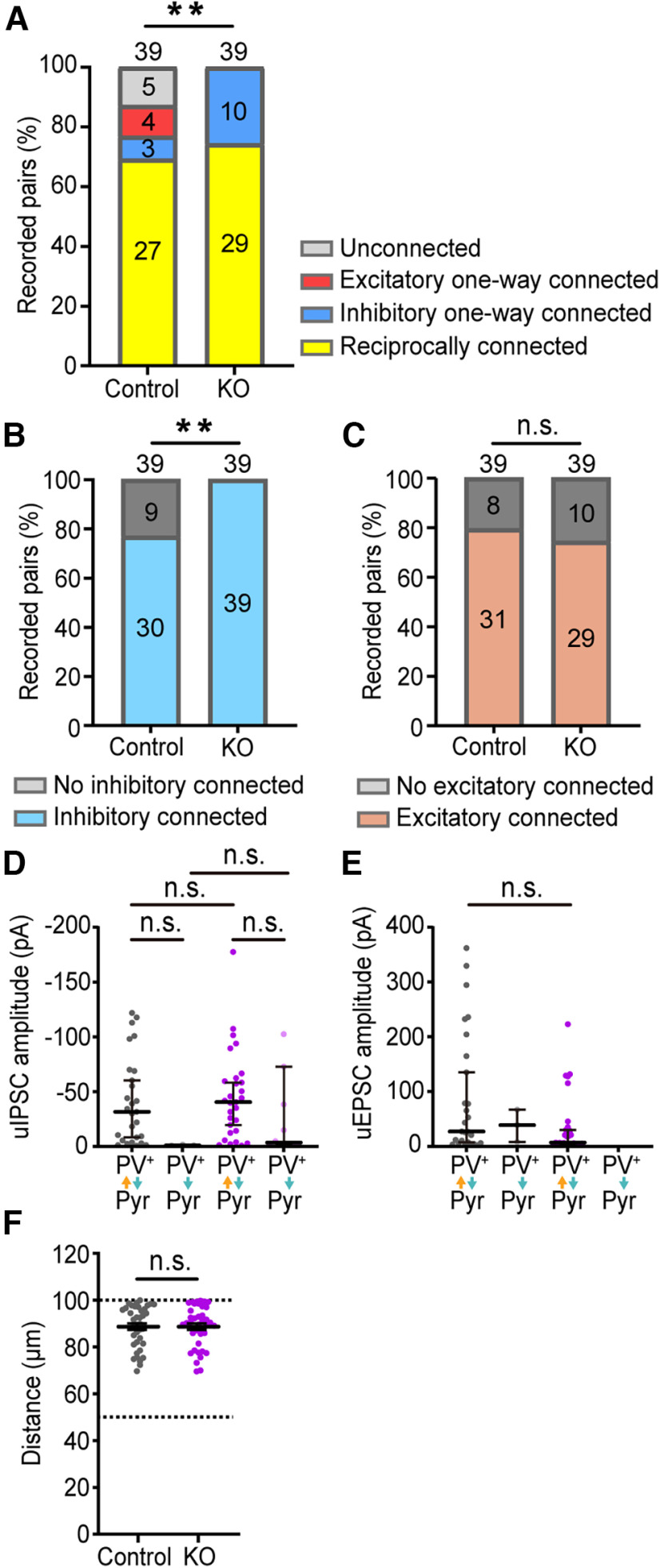
Synaptic connections between PV^+^ cell and Pyr cell in layer 2/3 of mouse V1 region between 50 and 100 μm. ***A***, All connection patterns between PV^+^ cells and Pyr cells in control and *PV-cKO* mice are shown. Fisher's exact test, *p* = 0.0027. ***B***, Same as ***A***, but ***B*** is inhibitory connection patterns. Fisher's exact test, *p* = 0.0023. ***C***, Same as ***A***, but ***C*** is excitatory connection patterns. Fisher's exact test, *p* = 0.7877. ***D***, Amplitude of the uIPSCs in ***A***. Bars indicate the median ± 95% CI value. Dunnett’s T3 multiple comparison test. *p *=* *0.0806 (cont. reciprocal vs cont. one-way), *p *=* *0.3164 (KO reciprocal vs KO one-way), *p *>* *0.9999 (cont. reciprocal vs KO reciprocal); *p *=* *9496 (cont. one-way vs one-way KO), *p *=* *0.7191 (cont. reciprocal vs KO one-way), and *p *=* *0.0413 (cont. one-way vs reciprocal KO). ***E***, Amplitude of uEPSCs between Pyr cell and PV^+^ cell in ***A***. Bars indicate the median ± 95% CI value. Dunnett’s T3 multiple comparison test; *p *>* *0.9999 (cont. reciprocal vs cont. one-way), *p *=* *0.1538 (cont. reciprocal vs KO reciprocal), *p *>* *0.9701 (cont. one-way vs reciprocal KO). ***F***, Distance between recorded PV^+^ cells and Pyr cell pairs in control and *PV-cKO* mice. Bars indicate the mean ± SEM value. Welch's *t* test. *p* = 0.9950. *p <* *0.05, **p <* *0.01; n.s. *p *>* *0.05.

Because the complexity of dendrites within 50–149 μm from the soma was increased in *PV-cKO* PV^+^ cells (Fig. 4*C*), next we performed additional simultaneous double whole-cell recordings from PV^+^ cells and Pyr cells located 50–100 μm apart (Fig. 6). It was found that the percentages of PV^+^ cells forming inhibitory synapses with Pyr cells were significantly increased in *PV-cKO* mice compared with control mice (Fig. 6*B*). However, the percentages of Pyr cells forming excitatory synapses onto PV^+^ cells were not different between control and *PV-cKO* mice (Fig. 6*C*). There were also no differences in the probabilities of reciprocal connection of PV^+^ cells and Pyr cells between control and *PV-cKO* mice (Fig. 6*A*). In both control and *PV-cKO* mice, the amplitudes of uIPSCs of reciprocal pairs tended to be higher than those of one-way pairs (Fig. 6*D*), but there was no significant difference in uIPSC amplitude between control and *PV-cKO* mice (Fig. 6*D*). The amplitudes uEPSCs in the reciprocal pairs were also not significantly different between the control and *PV-cKO* mice (Fig. 6*E*).

### The deletion of *cPcdhγ* in PV^+^ cells does not affect the apparent connection probability or the properties of synaptic responses between Pyr and PV^+^ cells below 50 **μ**m

**Figure 7.**
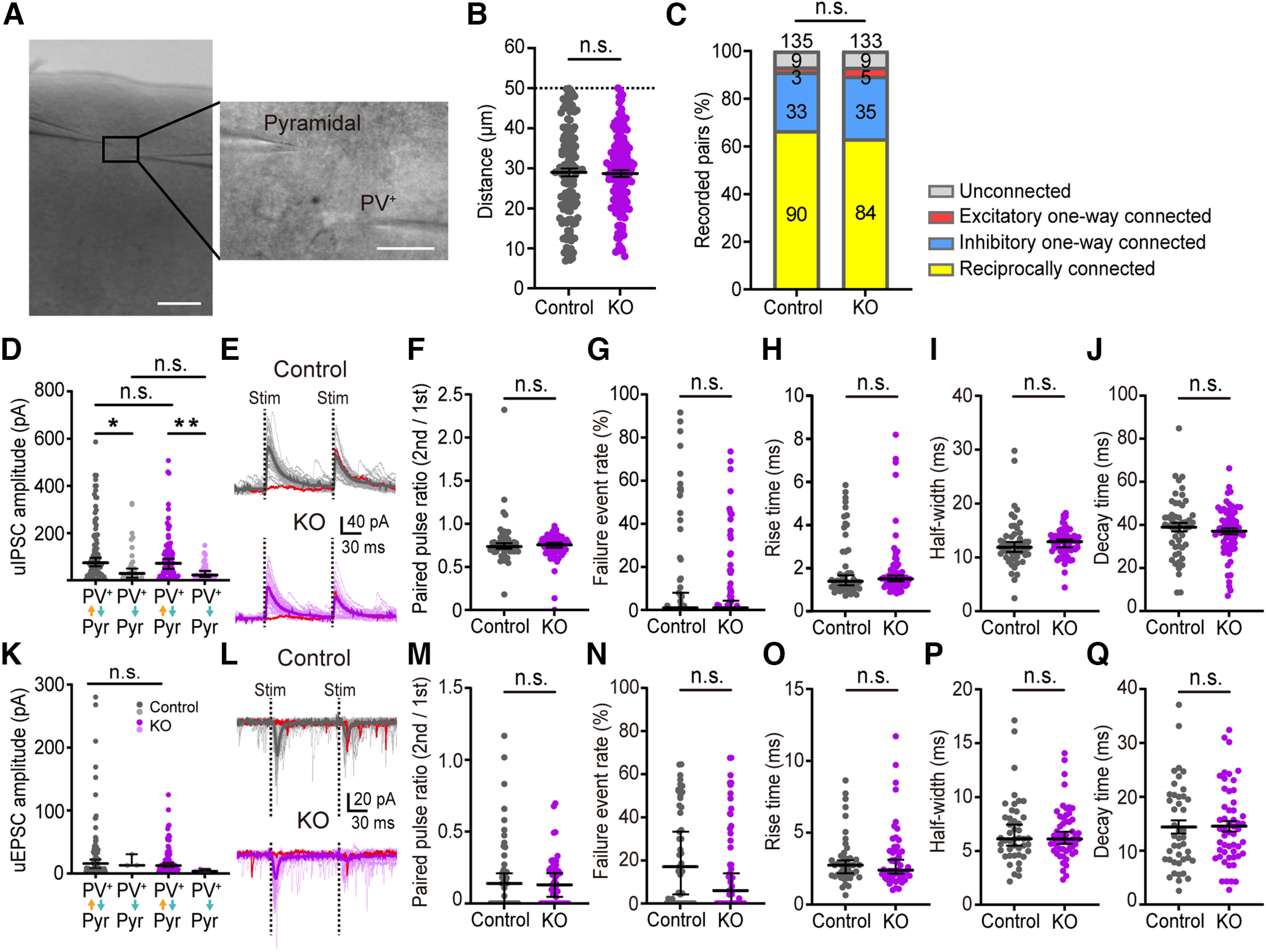
Synaptic connections between PV^+^ cell and Pyr cell in layer 2/3 of mouse V1 region. ***A*,** Image of a brain slice of the V1 region with two electrodes (left). Scale bar: 100 μm. High-magnification image of double whole-cell recordings (right). Scale bar: 20 μm. ***B***, Distance between recorded PV^+^ cells and Pyr cell pairs in control and *PV-cKO* mice. Bars indicate the mean ± SEM value. Welch’s *t* test. *p *=* *0.7410. ***C***, The probabilities of each connection pattern between PV^+^ cells and Pyr cells in control and *PV-cKO* mice are shown. χ^2^ test, *p *=* *0.8612. ***D***, Amplitude of the uIPSCs in ***C***. Bars indicate the median ± 95% CI value. Dunnett’s T3 multiple comparison test. *p *=* *0.0163 (cont. reciprocal vs cont. one-way), *p *=* *0.0038 (KO reciprocal vs KO one-way), *p *>* *0.9999 (cont. reciprocal vs KO reciprocal); *p *>* *0.9999 (cont. one-way vs one-way KO), *p *=* *0.0004 (cont. reciprocal vs KO one-way), and *p *=* *0.0889 (cont. one-way vs reciprocal KO). ***E***, Representative traces of uIPSC recorded between Pyr cells and PV^+^ cells in the control and *PV-cKO* mice. Fifty traces (light color), averaged traces (dark color), and a miss sweep (red) are shown. ***F***, Paired pulse ratio of uIPSC (control: *n* = 52 cells; KO: *n* = 68 cells). ***G***, Failure event rate of uIPSCs (control, *n* = 52 cells; KO, *n* = 68 cells). ***H***, Rise time of uIPSCs (control, *n* = 51 cells; KO, *n* = 66 cells). ***I***, Half-width of uIPSCs (control: *n* = 52 cells; KO: *n* = 68 cells). ***J***, Decay time of uIPSCs (control: *n* = 51 cells; KO: *n* = 67 cells). The bar indicates the median ± 95% CI value of (***F–I***). Mann–Whitney *U* test, *p *=* *0.8514 (***F***), *p *=* *0.5889 (***G***), *p *=* *0.4545 (***H***), *p *=* *0.1871 (***I***). The bar indicates the mean ± SEM value of ***J***. Welch’s *t* test, *p *=* *0.451 (***J***). ***K***, Amplitude of uEPSCs between Pyr cell and PV^+^ cell in ***C***. Bars indicate the median value ± 95% CI value. Dunnett’s T3 multiple comparison test. *p *>* *0.9999 (cont. reciprocal vs cont. one-way), *p *=* *0.1352 (KO reciprocal vs KO one-way), *p *>* *0.9999 (cont. reciprocal vs KO reciprocal); *p *=* *0.4003 (cont. one-way vs one-way KO), *p *=* *0.0803 (cont. reciprocal vs KO one-way); *p *>* *0.9999 (cont. one-way vs reciprocal KO). ***L***, similar as ***E***, but ***L*** is a representative trace of uEPSC. ***M***, Paired pulse ratio of uEPSC (control, *n* = 45 cells; KO, *n* = 54 cells). ***N***, Failure event rate of uEPSCs (control: *n* = 45 cells; KO: *n* = 54 cells). ***O***, Rise time of uIPSCs (control: *n* = 45 cells; KO: *n* = 53 cells). ***P***, Half-width of uEPSCs (control: *n* = 45 cells; KO: *n* = 54 cells). ***Q***, Decay time of uEPSCs (control: *n* = 43 cells; KO: *n* = 53 cells). The bar indicates the median ± 95% CI median value of ***M–P***. Mann–Whitney *U* test, *p *=* *0.9526 (***M***), *p *=* *0.1195 (***N***), *p *=* *0.9604 (***O***), *p *=* *0.9526 (***P***). The bars indicate the mean ± SEM value of ***Q***. Welch’s *t* test, *p *=* *0.934 (***Q***). *p <* *0.05, **p <* *0.01, *p <* *0.05; n.s. *p* > 0.05.

Our previous findings showed that in the barrel cortex cell-lineage-dependent reciprocal connections between excitatory neuron pairs with intracellular distance below 50 μm are significantly reduced in *cPcdh*-deficient neurons Tarusawa et al., 2016). Next, we conducted simultaneous double whole-cell recordings from PV^+^ cells and Pyr cells located within 50 μm of each other, to determine whether cPcdhγ is involved in the connectivity between PV^+^ cells and Pyr cells (Fig. 7*A*,*B*). In control mice, PV^+^ cells established inhibitory synapses with Pyr cells in 91% of the recorded pairs. Pyr cells formed excitatory synapses onto PV^+^ cells in 69% of cases, resulting in ∼67% of pairs being reciprocally connected. The percentage of excitatory one-way connected pairs was only 2% (Fig. 7*C*). These findings align with previous research (Hofer et al., 2011; D’Souza et al., 2019). The connectivity in PV^+^ cell specific *PV-cKO* mice was nearly identical to that in control mice (Fig. 7*C*). The impact of *cPcdhγ* deletion on the strength of synaptic connections was assessed by analyzing the amplitude of the synaptic responses between the two cells. Previous research has shown that the amplitude of unitary IPSCs (uIPSCs) is significantly greater in reciprocal pairs than in inhibitory one-way pairs (Yoshimura and Callaway, 2005). We also found a significant difference in the amplitude of uIPSCs between reciprocal pairs and inhibitory one-way pairs (Fig. 7*D*), both in control and *PV-cKO* mice. However, no significant difference in uIPSC amplitude was found between control and *PV-cKO* mice (Fig. 7*D*). The amplitudes of unitary EPSCs (uEPSCs) in the reciprocal pairs were also not significantly different between the control and *PV-cKO* mice (Fig. 7*K*). These results indicate that the deletion of *cPcdhγ* in PV^+^ cells does not influence the connection probability between PV^+^ cells and Pyr cells located within 50 μm of each other. Analysis of the waveform kinetics of the uIPSCs and uEPSCs showed that in both control and *PV-cKO* mice, the failure event rate, paired pulse ratio, half-width, rise time, and decay time of uIPSCs (Fig. 7*E–J*) and uEPSCs (Fig. 7*L–Q*) were similar. These results suggest that *cPcdhγ* in PV^+^ cells does not affect the properties of the synaptic responses at inhibitory and excitatory synapses.

### Individual PV^+^ cell-specific neural connectivity with Pyr cells was impaired by the deletion of *cPcdhγ* in PV^+^ cells

**Figure 8.**
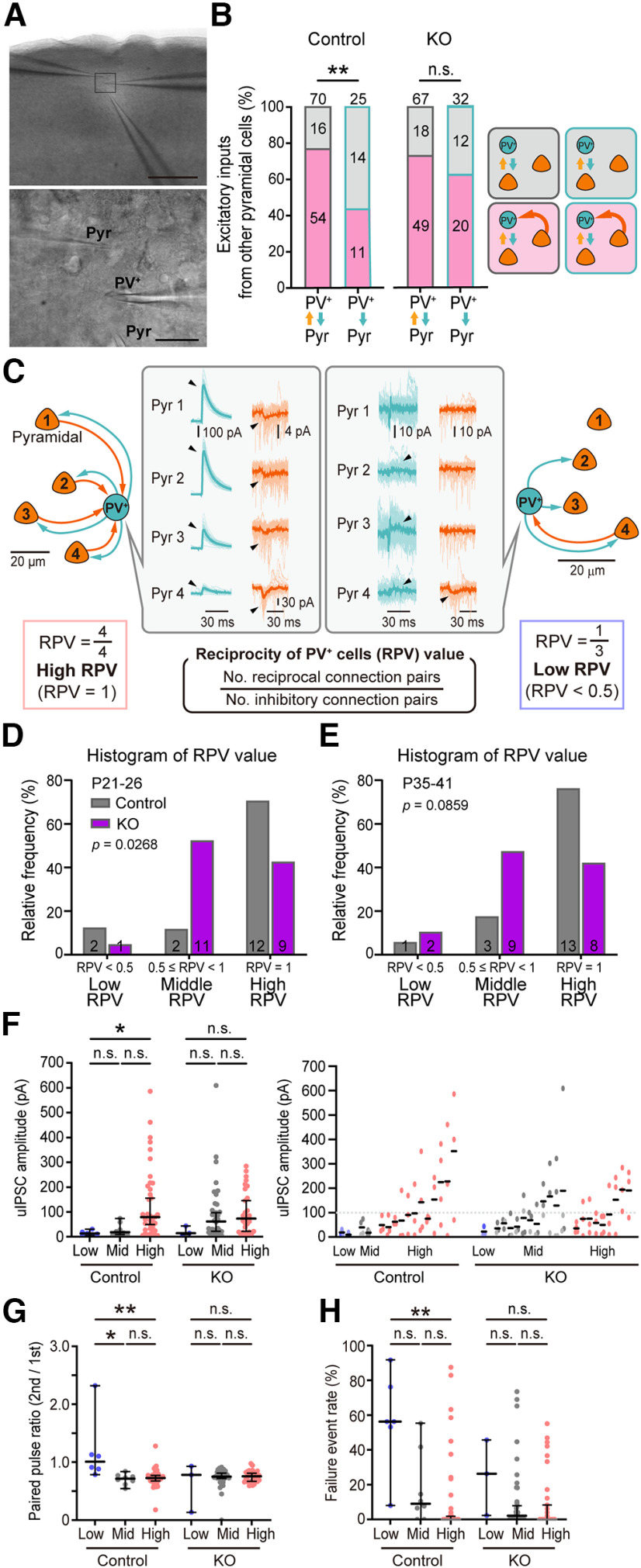
Deletion of *cPcdhγ* in PV^+^ cells affects the specificity of local neural connections between single PV^+^ cell and multiple Pyr cells. ***A***, Image of a brain slice with recording electrodes in the layer 2/3 V1 region (upper). Scale bar: 200 μm. High-magnification images of triple whole-cell recordings (lower panel). Scale bar: 20 μm. ***B***, Right, The Probability of excitatory inputs from a third party of Pyr cells on PV^+^ cells with different connectivity. Left, Schematic illustration of three-cell connection relationship. Fisher’s exact test. *p *=* *0.0049 (control: reciprocal vs one-way) and *p *=* *0.3508 (KO: reciprocal vs one-way). ***C***, Two examples of actual connectivity between a single PV^+^ cell and multiple Pyr cells. Unitary synaptic responses of 50 trials (light color) and averaged traces (dark color) are shown. The reciprocity of PV^+^ cells (RPV) is the number of reciprocally connected pairs divided by the number of inhibitory connected pairs. ***D***, Classification of PV^+^ cells by connectivity with multiple Pyr cells in P21–P26 (low RPV: RPV value 0.3333; middle RPV: RPV value 0.5–0.75; high RPV: RPV value 1.0). Fisher’s exact test. *p *=* *0.0268. Control: *n* = 16 PV^+^ cells; *PV-cKO*: *n* = 21 PV^+^ cells. ***E***, The same as ***D***, but ***E*** is in P35–P41. Fisher’s exact test. *p *=* *0.0859. Control: *n* = 17 PV^+^ cells; KO: *n* = 19 PV^+^ cells. ***F***, Left, Amplitude of uIPSC in each RPV. Dunn’s multiple comparisons test. *p *>* *0.9999 (cont. Low vs cont. Mid); *p *=* *0.212 (cont. Mid vs cont. High); *p *>* *0.0031 (cont. Low vs cont. High), *p *>* *0.9999 (KO Low vs KO Mid), *p *>* *0.9999 (KO Mid vs KO High), *p *>* *0.9999 (KO Low vs KO High). Right, uIPSCs plotted for each PV^+^ cell. The light colors of the plots represent one-way connection pairs. ***G***, Paired pulse ratio of the uIPSC. Dunn’s multiple comparisons test; *p *=* *0.0136 (cont. Low vs cont. mid), *p *>* *0.9999 (cont. Mid vs cont. High); *p *>* *0.0243 (cont. Low vs cont. High), *p *>* *0.9999 (KO Low vs KO Mid), *p *>* *0.9999 (KO Mid vs KO High), *p *>* *0.9999 (KO Low vs KO High), *p *=* *0.0329 (cont. Low vs KO Mid), and *p *=* *0.0243 (cont. Low vs KO High). ***H***, Failure event rate of uEPSC. Dunn’s multiple comparisons test. *p *=* *0.9058 (cont. Low vs cont. mid), *p *>* *0.9999 (cont. Mid vs cont. High; *p *=* *0.0019; cont. Low vs cont. High), *p *>* *0.9999 (KO Low vs KO Mid), *p *>* *0.9999 (KO Mid vs KO High), *p *>* *0.9999 (KO Low vs KO High), *p *=* *0.014 (cont. Low vs KO Mid), and *p *=* *0.0046 (cont. Low vs KO High); *p <* *0.05, **p <* *0.01, n.s. *p *>* *0.05. Bars indicate the median ± 95% CI value.

Previous reports have demonstrated that PV^+^ cells form synapses onto Pyr cells nonspecifically (Packer and Yuste, 2011). However, the converse is not true; Pyr cells can selectively target PV^+^ cells (Yoshimura and Callaway, 2005). We further examined the connectivity of each PV^+^ cell with multiple Pyr cells. We performed simultaneous whole-cell patch clamp recordings from a single PV^+^ cell and two Pyr cells (Fig. 8*A*,*B*). In control mice, we found that reciprocally connected PV^+^ cells received significantly more inputs from other Pyr cells compared with PV^+^ cells connected in a one-way inhibitory fashion (Fig. 8*B*). These results indicate the two patterns of connectivity between PV^+^ cells and Pyr cells: PV^+^ cells that preferentially receive inputs from multiple Pyr cells, and PV^+^ cells that receive fewer inputs from Pyr cells. Strikingly, this preference for excitatory inputs from Pyr cells disappeared in the *cPcdhγ-cKO* mice. To quantify the bias in the inputs from the Pyr cells to individual PV^+^ cells, we examined the connectivity between a single PV^+^ cell and surrounding >3 Pyr cells (Fig. 8*C*). The connectivity of each PV^+^ cells with multiple Pyr cells was categorized. We calculated the connectivity of individual PV^+^ cells as the reciprocity of PV^+^ cells (RPV): the number of reciprocally connected pairs divided by the number of pairs that bind at inhibitory synapses (Fig. 8*C*). An RPV value of 1 indicates that all Pyr cells that receive inhibitory inputs from the PV^+^ cell are connected reciprocally. We categorized PV^+^ cells into three groups according to the RPV value: low-RPV (0 < RPV < 0.5), middle RPV (0.5 ≦ RPV < 1), and high-RPV (RPV = 1). We found that 75% of PV^+^ cells were categorized as having high-RPV in control mice, whereas the remaining PV^+^ cells were equally distributed in the low or middle RPV (Fig. 8*D*) at P21–P26. In contrast, *PV-cKO* mice showed that middle RPV was most common (52%), and the proportion of high-RPV was reduced to 43% (Fig. 8*D*). PV^+^ cells in the low-RPV group showed significantly lower amplitude of uIPSCs and higher paired pulse ratio and failure rate compared with PV^+^ cells in the middle and high-RPV groups in control mice (Fig. 8*F–H*). Contrarily, PV^+^ cells in the low-RPV group in *PV-cKO* mice, did not exhibit the characteristics of the low-RPV group in control mice. The amplitude of uIPSCs in the middle RPV in cKO mice was similar to that of high-RPV in control mice (Fig. 8*F*), indicating that high-RPV might become a middle RPV by the reduction of reciprocity in *PV-cKO* mice. Similar to those at P21–P26, different distributions of high-RPV, middle-RPV, and low-RPV between control and *PV-cKO* mice appeared at P35–P41 adult stages (Fig. 8*E*). These results suggest that *cPcdhγ* determines the characteristics of excitatory synaptic partners in each PV^+^ cell.

## Discussion

**Figure 9.**
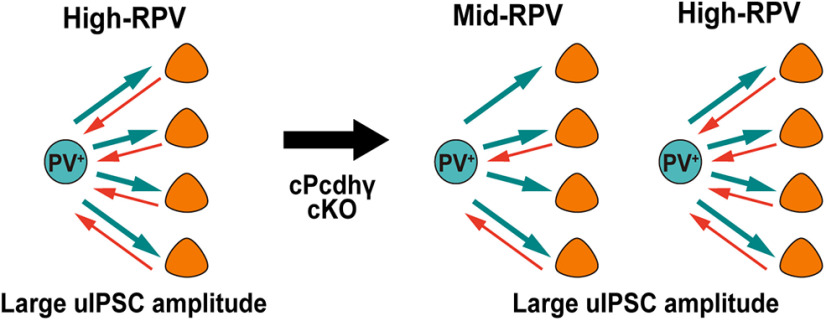
Effects of PV^+^ cell-specific cPcdhγ KO on neural circuit formation in mouse primary visual cortex.

We evaluated the effects of *cPcdhγ* deletion in PV^+^ cells on the formation of neural circuits in the visual cortex. We chose a developmental time point of *cPcdhγ* deletion when peak programmed cell death has passed. Here, we found that connectivity of PV^+^-Pyr was significantly different between control and *PV-cKO* mice. Reciprocal connected PV^+^ cells to Pyr cells were significantly higher than one way connected PV^+^ cells in control mice. However, there were no significant differences in *PV-cKO* mice. The proportion of high reciprocal connected PV^+^ cells to Pyr cells with large uIPSC amplitudes was reduced in *PV-cKO* mice, and the reciprocity of PV^+^ cells connected to Pyr cells with large uIPSC amplitudes was reduced (Fig. 9). These results indicated that *cPcdhγ* in PV^+^ cells regulates their reciprocity with Pyr cells in the cortex. We demonstrated for the first time the function of *cPcdhγ* in inhibitory neurons in neural circuit formation.

### Influence of deletion of *cPcdhγ* in PV^+^ cells on the dendritic morphology of PV^+^ cells

Expression of *cPcdhγ* is essential for dendritic formation in several neurons. Deletion of *cPcdhγ* in Purkinje and starburst amacrine cells abrogates repulsion and causes self-recognition and increased self-crossing (Lefebvre et al., 2012). In excitatory neurons of layer 5 in the somatosensory cortex, dendritic complexity is reduced by *cPcdhγ* deletion (Garrett et al., 2012; Molumby et al., 2016). Our results also revealed abnormal dendritic formation in PV^+^ cells, evidenced by increased dendritic complexity between 100–149 μm from the soma and a reduction of longer dendrites because of *cPcdhγ* deletion (Fig. 4). Therefore, it seems *cPcdhγ* isoforms regulate dendrite formation in PV^+^ cells as well.

### Influence of *cPcdhγ* deletion in PV^+^ cells on the cortical neural circuits

Deletion of *cPcdhγ* in inhibitory neurons during the early stages of cortical development caused neural cell death (Carriere et al., 2020; Leon et al., 2020). In particular, deletion of *cPcdhγ* in inhibitory neurons leads to excessive cell death in the cortex from around P8 (Carriere et al., 2020). Programmed cell death of inhibitory cells is regulated by excitatory inputs from Pyr cells, indicating that *cPcdhγ* may be involved in neural circuit formation (Wong et al., 2018). Here, using PV^+^-specific *cPcdhγ*-deficient mice, we could examine the function of *cPcdhγ* in neural circuit formation without causing cell death. Through laser-scan photostimulation of caged glutamate, no significant differences were observed in the excitatory input source and input strength of PV^+^ cells in layer 2/3 between control and *PV-cKO* mice by Bonferroni’s multiple comparisons test in Figure 5. Conversely, multiple whole-cell recordings of single PV^+^ cells to multiple Pyr cells revealed two distinct types of PV^+^ cells: high-RPV with large uIPSC, and middle-RPV and low-RPV with small uIPSC in 2/3 layer of the visual cortex in control mice. However, in *PV-cKO* mice, the proportion of high-RPV with large uIPSC decreased, and the proportion of middle-RPV with large uIPSC increased, suggesting that the reciprocity of PV^+^-Pyr connections is reduced in *PV-cKO* mice. This proportional abnormality in *PV-cKO* mice was initially observed at P21–P26 and was also observed at P35–P41 in the adult stage, suggesting that it is not caused by developmental delay in neural circuit maturation. In retinal starburst amacrine cells, *cPcdhγ* is required for synapse elimination during development (Kostadinov and Sanes, 2015), indicating the possibility that the reduction of reciprocal connections between PV^+^ cells and Pyr cells in *PV-cKO* mice may be because of impaired elimination of excitatory synapses during circuit development before P21. Interestingly, the ratio of reciprocal connectivity between clonal layer four excitatory cells in the barrel cortex increases from P9–P11 to P13–P16 in wild-type cells, but this increase does not occur in *cPcdh*-deficient cells. Additionally, further elimination of one-way connectivity is observed from P13–P16 to P18–P20 in wild-type cells, but this elimination does not occur in *cPcdh*-deficient cells (Tarusawa et al., 2016). Future analysis of PV^+^-Pyr connectivity early in life is needed to determine whether elimination is occurring.

### Functional meaning of different reciprocal connectivity of PV^+^ cells

As discussed above, we made a novel discovery that PV^+^ cells can be categorized into different types in control mice: high-RPV with large uIPSC and low-RPV or middle-RPV with small uIPSC. While there are no reports on the proportion of reciprocal connections of PV^+^ cells from multiple Pyr cells, it has been documented that several types of basket cells exist based on mRNA expression and cell morphology (Gouwens et al., 2019), and that each basket cell has different visual response characteristics (Hofer et al., 2011) and dendritic morphologies (Runyan and Sur, 2013). Recently, it has also been reported that certain PV^+^ cells show different rates of reciprocal connections in other brain regions. Therefore, the functional and morphologic features of these two different types of PV^+^ cells in relation to reciprocity and uIPSC amplitude observed in control mice need to be examined in future studies.

Elaborating functional significance of PV^+^ cell types is beyond the scope of this study; however, two plausible hypotheses can be proposed. One hypothesis involves a preference for visual stimulus response. Excitatory cells in the primary visual cortex exhibit high orientation selectivity, responding only to visual stimuli of a specific orientation. However, only ∼18% of PV^+^ cells show a high degree of orientation selectivity, while most PV^+^ cells are active in response to visual stimuli of any orientation (Hofer et al., 2011). This observation aligns with the results of our study, where 25% and 23% of low-RPV or middle-RPV cells were observed in P21–P26 and P35–P41, respectively, in control mice. Each Pyr cell has varying orientation selectivity. PV^+^ cells that are reciprocally connected to many Pyr cells (high-RPV) can respond to multiple orientations and become less orientation-selective because of inputs from numerous Pyr cells. In contrast, PV^+^ cells with reciprocal connections to only a few Pyr cells (low-RPV) may exhibit biased orientation selectivity.

The second hypothesis involves the thalamic input. Relay neurons in the lateral geniculate nucleus are mainly projected to L4 of the visual cortex, but there are also patchy projection areas in L1. In PV^+^-Pyr pairs in L2/3 under the patchy projection areas, the Pyr cells receive significantly smaller uIPSC amplitudes compared with the pairs under the interpatch, despite no difference in the connection probability of reciprocity (D’Souza et al., 2019). We found that PV^+^ cells within different types of local neural circuits significantly differ in terms of the rate of reciprocal connections and uIPSC amplitudes, suggesting a potential link between thalamic input and the location of PV^+^ cells with high-RPV and low-RPV or middle-RPV characteristics.

### Molecular mechanisms of regulation of neural circuit formation by *cPcdhγ*

The increase of dendritic complexity near the PV^+^ cell soma (50–200 μm) in *PV-cKO* mice may contribute to the increase in excitatory inputs, as the probability of PV^+^ cell dendrites encountering axons of Pyr cells is increased. The probability of inhibitory synaptic connections within 50 μm of PV^+^ cells was not affected by *cPcdhγ* deletion; however, excitatory synapses were affected (Fig. 8), which aligns with the notion that PV^+^ cells target adjacent Pyr cells nonspecifically (Packer and Yuste, 2011), while Pyr cells selectively target their binding partners (Yoshimura and Callaway, 2005). In this study, the probability of inhibitory synaptic connections from PV^+^ cells located 50–100 μm away from Pyr cells was significantly increased in *cPcdhγ* deletion (Fig. 6*A*), suggesting that *cPcdhγ* may also regulate inhibitory synaptic connections from more distant PV^+^ cells. Interestingly, the targeting probability from PV^+^ cells to Pyr cells decreases with distance (Packer and Yuste, 2011). However, our results only examined the role of *cPcdhγ* in PV^+^ cells on PV^+^-Pyr connectivity. Since *cPcdhγ* is also expressed in Pyr cells, it is important to analyze *cPcdhγ-cKO* Pyr cells to further understand the molecular mechanisms of transinteractions of cPcdhγ proteins in synaptic connectivity.

## Synthesis

Reviewing Editor: Matthew Grubb, King’s College London Decisions are customarily a result of the Reviewing Editor and the peer reviewers coming together and discussing their recommendations until a consensus is reached. When revisions are invited, a fact-based synthesis statement explaining their decision and outlining what is needed to prepare a revision will be listed below. The following reviewer(s) agreed to reveal their identity: NONE. Note: If this manuscript was transferred from JNeurosci and a decision was made to accept the manuscript without peer review, a brief statement to this effect will instead be what is listed below.

Synthesis:

Both previous JN Reviewers found the revised manuscript to be much improved but still with significant issues that require resolution. Reviewer 1 suggests adding more data for Fig 4, and this is an option, but I do not think it is absolutely necessary. Reviewer 2 suggests some very clear revisions to the description and presentation of the data in Fig4 which, if implemented in full, should address these issues in a satisfactory manner. Please also address, in full, all of the other Reviewers’ comments.

Reviewer 1:

In the revised version, the quality of the paper was clearly improved. However, there are still a few important issues left.

Comments:

(1) There is an inconsistency in describing the results of uncaging experiments in fig. 4. In the rebuttal letter, the authors said ‘our analysis ... in Figure 4A-H revealed no elevation in input from the vicinity of the PV neurons.’, while the paper described the results differently ‘The cPcdhγ-deleted PV+ cells appeared to receive larger mean amplitudes and more numbers of photostimulation-evoked EPSCs events’ and ‘there appeared to be a slight increase in input’.

(2) More importantly, what is the answer to the question ‘do PV neurons in KO really receive stronger excitation from the neighborhood’? I am glad to see more data points were added to the figure. However, most of them had smaller amplitude and less number of events than the old data points and they made the difference in excitation received by PV cells between control and KO diminished. The distribution of newly added data points is obviously deviated from that of the old data. More recording is needed to give a solid answer.

(3) Because the above two points, I don’t think the second and third points in previous comments have been properly addressed.

(4) The axonal morphology of PV cells should be shown in figures to demonstrate that they are basket cells, but not chandelier cells.

Reviewer 2:

The revised manuscript is significantly improved and includes new experiments. The authors addressed most of the previous concerns. Overall, the revised study supports a role for the Pcdhgs in refining the reciprocal connectivity patterns between Pyramidal neurons and nearby inhibitory interneurons. The uncaging and paired recordings are elegant approaches. The findings are interesting and important, and add to a growing body of studies implicating the Pcdhgs in influencing synaptic pairing between nearby cells. The findings that Pcdhg deletion does not affect the overall membrane and synaptic properties of PV cells is also important. However, new data that now diminish the previous claims that excitatory inputs from proximal Pyramidal cells onto PcdhgcKO PV+ cells were elevated (Fig4) are reported in a confusing manner. The overall logic of the experiments and the chosen Pyr-PV distances are not clear, and not clearly connected to the more important findings relating to RPV.

The following concerns should be clarified.

1. The revised data in Fig 4 now indicate that there are fewer significant differences in Pyramidal inputs onto cPcdhγ-deleted PV+ cells, though some trends of elevated connections remain.

In the rebuttal, the authors state’ Our analysis, incorporating an increased sample size in Figure 4A-H, revealed no elevation in input from the vicinity of the PV neurons. This finding eliminates the discrepancy present in the revised manuscript.’. But the reporting of these data are confusing, as they alternate between emphasizing or diminishing the importance of elevated connections. The authors should carefully review the data (which are highly variable) and report in an accurate but coherent manner. For example:

a. There are overstatements such as this sentence describing data in Fig. 4E, F:

“The cPcdhγ-deleted PV+ cells appeared to receive larger mean amplitudes and more numbers of photostimulation-evoked EPSCs events only in L2/3 stimulation but not significantly different (Fig. 4E,F)”.

-> The data do not justify this statement. The plot shows a broader distribution of measurements, leading to a small increase in the mean for cPcdhγ-deleted PV+ cells, but many measurements are in the lower end, as in control.

b. “As a result, there appeared to be a slight increase in input, especially from Pyr cells 50-180 μm away (only significantly different in 150 and 180 μm away) (Fig. 4H-I), suggesting that cPcdhγ in PV+ cells may be involved in the formation of synaptic connections with surrounding Pyr cells.”

->The difference is very subtle, and cannot be interpreted as meaningful to ‘the formation of synaptic connections with surrounding Pyr cells’. It would be useful to include a cumulative statistic to compare the two populations. KS statistics in the table indicate that they don’t differ significantly; but the KS is not appropriate here for comparing longitudinal data over distance.

c. It is not clear if this statement in the discussion is referring to Fig 4 and/or 7A?: The probability of inhibitory synaptic connections within 50 μm of PV+ cells was not affected by cPcdhγ deletion; however, excitatory synapses were affected, which aligns with the notion that PV+ cells target adjacent Pyr cells non-specifically (Packer and Yuste, 2011).

d. the title for Figure 4 should be changed to one that aligns with the current assessment.

2. The data in Fig4 are still likely underpowered. This limitation should be raised in the discussion. At this point, the rate of Pyr-PV connectivity along different distances is inconclusive, and it’s premature to state ‘no elevation in input from the vicinity of the PV neurons’. The PcdhgcKO PV responses are much more variable - is this noteworthy?

3. Regardless of Fig 4, the RPV analyses are the stronger findings and the model can be drawn independently (and might suggest reduced probability of Pyr-PV connectivity?). The logic of the experiments and the findings should be more clearly laid out and reconciled.

4. In the new Figure 7, the authors analyzed Pyr-PV connections within a 50-100um distance. What is the rationale for focusing on this range, as opposed to 150-180? They state in the text ‘ Laser-scan photostimulation experiments indicated that cPcdhγ in PV+ cells received slightly more excitatory inputs from surrounding Pry cells located 50-100 μm away from recording PV+ cells.” Does this refer to data in Figure 4, which are not conclusive? Fig. 4H, 4I suggested ‘slightly more inputs’ in the 150-180um ranges. The authors should clearly explain the logic of the experiments and the selected distances. This section could also benefit from a few lines that establish how the authors interpret the importance of Pyr-PV distances in their phenotypes.

5. The introduction still omits important work on the cPcdhs, and should be carefully reviewed by the authors

For example, Line 95+ should cite: It is known that cPcdhs have homophilic binding properties, suggesting their involvement in differentiating between self and other neurons (Esumi et al., 2005; Kaneko et al., 2006; Hirano et al., 2012; Yagi,2012).

-> Lefebvre 2012; Schreiner and Weiner PNAS; Mountofaris 2017 who showed cPcdha diversity in olfactory sensory neurons; key Shapiro and Honig papers, ie Brasch 2019, Rubinstein 2017; Goodman eLife 2022

6. This statement should be nuanced, as cPcdh expression varies by neuronal cell type (as shown by authors own work on Serotonergic cells, and sequencing by Chen et al 2017)

‘Each neuron expresses its own set of approximately 15 isoforms’. The authors should cite Lv et al for the Pyramidal cell expression.

Carriere et al also showed no change PV-Cre; Pcdhg animals: Line 312: Similar cell number ... consistent with the previous study of Leon et al. (Leon et al., 2020).

There are several typos throughout the text and in the figures:

Line 37: clustered protocadherinγ (cPcdhγ) gene deletion: change clustered protocadherin gamma

Line 395: ‘Pry’ should be Pyr ; ‘excitatory inputs from surrounding Pry cells located 50-100 μm ...’

657 signals. Scale bar: 20 μm. C, Same as B, bu representing image of cPcdhγ (red) and vGluT1 (green)

Line 662: For Figure legend 2D, Specify that cPcdhγ mRNA signal is (green)

...’Low-magnification images of smFISH of PV mRNA (magenta) and cPcdhγ mRNA [add green] encoding constant

Figure 5B: ‘Numeber’ typo

Figure 9: ‘connecion’ should be connection

Line 311: replace ‘disappeared’ with undetectable

Finally, I would like to extend my sincere condolences for the loss of your colleague, Dr. Tarusawa.
